# Functional near-infrared spectroscopy: Systematic mapping of abnormal brain function features in neurological disorders

**DOI:** 10.4103/NRR.NRR-D-25-00595

**Published:** 2026-01-02

**Authors:** Yunjie Li, Yangyang Feng, Xia Liu, Ruochao Yuan, Shiling Chen, Jingyi Wang, Chao Pan, Gaigai Li, Zhouping Tang

**Affiliations:** 1 Division of Child Healthcare, Department of Pediatrics, Tongji Hospital, Tongji Medical College, Huazhong University of Science and Technology, Wuhan, Hubei Province, China; 2 Department of Neurology, Jingzhou Hospital, Yangtze University, Jingzhou, Hubei Province, China; 3 Hubei Provincial Engineering Research Center of Neuromodulation Technology, Hubei Women’s and children’s Activity Center, Wuhan, Hubei Province, China; 4 Department of Neurology, Tongji Hospital, Tongji Medical College, Huazhong University of Science and Technology, Wuhan, Hubei Province, China

**Keywords:** biomarkers, brain mapping, clinical translational application, functional connectivity, functional near-infrared spectroscopy, multimodal fusion, nervous system diseases, neuroimaging, neuroregenerative therapy, neurovascular coupling

## Abstract

Functional near-infrared spectroscopy quantifies cerebral hemodynamic signals by capturing oxygenation-dependent changes in hemoglobin in a noninvasive, portable, and ecologically valid manner, providing a unique insight into neurovascular coupling. However, functional imaging biomarkers with high ecological validity for neurological disorders such as stroke, Parkinson’s disease, dementia, amyotrophic lateral sclerosis, epilepsy, spinal cord injury, and traumatic brain injury are lacking, limiting the mechanistic understanding, treatment evaluations, and individualized interventions. The aim of this review is to systematically summarize evidence from the past decade on the use of functional near-infrared spectroscopy under the aforementioned conditions, synthesize its value for revealing neural mechanisms and assessing therapeutic responses, and identify current technical bottlenecks and future directions for advancement. Collectively, the findings demonstrate that functional near-infrared spectroscopy possesses substantial and far-reaching potential for uncovering the neural mechanisms underlying disease and for evaluating treatment-induced changes in brain function. Equipped with wearable probes, functional near-infrared spectroscopy can continuously and noninvasively monitor brain activity in naturalistic environments for extended periods, thereby overcoming the limitations of conventional imaging modalities that can only acquire data under restricted settings. This capability can furnish unprecedented objective neuroimaging evidence for neuroregenerative therapy research. Moreover, the portability of functional near-infrared spectroscopy allows it to be integrated into neurofeedback training systems: hemoglobin signals can be fed back to participants within milliseconds, enabling targeted, individualized, closed-loop modulation of brain function and considerably expanding the scope of hemodynamics-based neurofeedback. When combined with other brain function assays (such as electroencephalography) and intervention techniques (such as transcranial magnetic stimulation and transcranial direct current stimulation), functional near-infrared spectroscopy also supplies high-temporal-resolution hemodynamic information, laying a critical foundation for the construction of high-precision noninvasive brain–computer interfaces, real-time cognitive-state decoding, and adaptive neuromodulation. Admittedly, almost all existing functional near-infrared spectroscopy studies are still observational and have small sample sizes, short follow-ups, and insufficient controls—shortcomings that together produce low-grade evidence. Therefore, there is still a significant gap before clinical translation can be achieved. Technically, the limited penetration depth of functional near-infrared spectroscopy restricts sampling to the superficial cortex, leaving deep nuclei largely unreachable. In addition, no consensus exists across devices regarding optode layout, light-source choice, motion-artifact correction, or analytical pipelines, creating pronounced heterogeneity that undermines reproducibility. With artificial intelligence and big data analytics advancing rapidly, functional near-infrared spectroscopy embedded within multimodal fusion frameworks is now poised to systematically map aberrant brain function signatures of neurological disorders, identify pathological regions suitable for targeted intervention, and provide real-time assessments of functional changes produced by neuroregenerative therapies.

**Facts**

• The human brain’s nervous system is extremely complex, and this complexity poses significant challenges for the accurate diagnosis and treatment of neurological diseases.

• Neurovascular coupling is the basis of functional imaging; therefore, monitoring changes in cerebral blood flow can infer the state of brain function.

• Traditional neuroimaging tools have significant limitations and cannot simultaneously meet the demands of naturalistic settings, high temporal resolution, and low cost, which restricts researchers’ comprehensive understanding of brain function.

**Open questions**

• How can hardware innovations overcome the limitations of functional near-infrared spectroscopy in terms of detection depth?

• Is it possible to establish a standardized paradigm across centers to validate the reliability of functional near-infrared spectroscopy-derived biomarkers in neurological injury and regeneration?

• Can functional near-infrared spectroscopy be integrated with other modalities to promote the clinical translational research of neuroregenerative therapies?

## Introduction

The nervous system is undoubtedly one of the most complex structures, containing 10^11^ neurons and four times as many glial cells, which together form 10^14^ synaptic connections (Pakkenberg et al., 2003; Azevedo et al., 2009). Understanding the complex functions and structures of the human brain, which are essential for the accurate diagnosis and treatment of neurological disorders, is highly challenging (Zlokovic, 2008; Khalil et al., 2024). Due to the lack of fast and high-resolution measurement tools in natural settings, our understanding of brain function has historically been too one-sided and rigid, often focusing solely on the functional ensembles of neurons while ignoring the important roles of glial cells and blood supply (Zlokovic, 2008). The nutrient supply within the brain depends on the continuous delivery of blood flow. When neural activity increases, the blood supply to neurons inevitably rises, suggesting that changes in brain activity can be inferred by calculating cerebral blood flow. This association is known as neurovascular coupling (Logothetis, 2002; Drew, 2022; Holstein-Ronsbo et al., 2023; Owens et al., 2024). Although the mechanisms of neurovascular coupling are not yet fully understood, scientists have sought to develop various imaging devices to study functional connectivity (FC) and brain activity based on hemodynamic changes under diverse conditions and research paradigms. However, conventional neuroimaging tools fail to simultaneously meet the demands of naturalistic settings, high temporal resolution, and cost-effective portability. For example, functional magnetic resonance imaging (fMRI) enables whole-brain imaging but is expensive, excludes certain populations, and is limited by its low-speed sampling of 1–2 seconds. Electroencephalography (EEG) and magnetoencephalography (MEG) have poor spatial resolution, while positron emission tomography (PET) involves exposure to strong radiation (Anzellotti et al., 2016; Li et al., 2019; van der Meulen et al., 2021).

**Figure 1:**
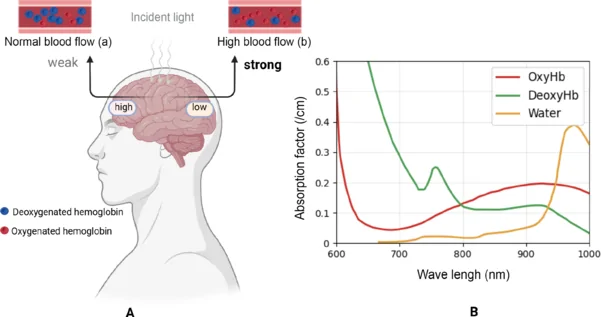
Schematic diagram of the working mechanism of near-infrared brain functional imaging. (A) Changes in local cerebral blood flow and oxygenation levels under two physiological states: rest (a) and activation (b). (B) The near-infrared light absorption spectra of the main light-absorbing components in blood. The absorbance data for water were taken from Kou, Labrie, and Chylek (1993) at https://omlc.org/spectra/water/, where the absorption coefficient per centimeter is provided directly. The hemoglobin (HbO_2_ and HbR) absorbance data were obtained from Scott Prahl at https://omlc.org/spectra/hemoglobin/; these data are given as molar extinction coefficients and were converted to the absorption coefficient per centimeter with the formula supplied in the dataset: µa = (2.303) ε (x g/L)/(64,500 g Hb/M). The blood volume fraction of the brain cortical is approximately 3% (Ito et al., 2001), and the water content of cortical gray matter is approximately 80% (Abbas et al., 2015). The absorbance values of water and hemoglobin were scaled accordingly based on these data. HbO_2_: Oxyhemoglobin; HbR: deoxyhemoglobin.

fNIRS is a novel optical imaging technique that uses light in the near-infrared range (NIR) with specific wavelengths to monitor changes in the absorbance of blood in brain tissue, which in turn reflects changes in brain activity (Almajidy et al., 2020; Zhao et al., 2023). The absorbance of NIR light projected onto the surface of brain tissue is calculated to assess brain activation and FC based on optical properties, serving as the working principle of NIR light in functional brain imaging (**Figure 1**; Almajidy et al., 2020; Mavileti et al., 2024). fNIRS has become one of the most cutting-edge research tools for understanding the human brain and is widely used in brain science and medical research, providing a completely new perspective for observing the brain. It is applied in various fields, including public health, safety, the military, humanities, sports, and in the medical domain, where it is extensively used in psychology, rehabilitation, geriatric health, neuroscience, otolaryngology, and pediatrics (Almajidy et al., 2020). Although fNIRS has been extensively explored in the context of neurological disorders, current research still has significant limitations. For instance, most studies primarily utilize fNIRS to investigate phenomena or characterize differences but lack in-depth explorations of the underlying physiological and pathological processes (Iadecola, 2017). Furthermore, few long-term observational studies have focused on neural injury and repair (Lorenz et al., 2024). Additionally, there is substantial heterogeneity in parameter settings, experimental paradigms, and data analysis methods across fNIRS studies (Stute et al., 2025).

Based on the above discussion, the aim of this review is to comprehensively examine the current applications of fNIRS in neurological disorders, such as epilepsy, amyotrophic lateral sclerosis (ALS), dementia, Parkinson’s disease (PD), stroke, and brain injury, while exploring its potential value in elucidating the mechanisms of neural injury, repair, and regeneration. Our review offers two innovations. First, we extract disease-specific fNIRS application parameters from the existing literature to provide a referenceable protocol for clinical trials. Second, we conduct a horizontal comparison of the application scenarios of different brain imaging modalities to investigate whether future multimodal fusion (e.g., combined EEG or fMRI) can improve the parsing accuracy of brain signals, thereby enhancing the reliability of fNIRS in neuroregeneration research.

## Search Strategy

Based on the search objective, the search strategy for this review was as follows: (1) Database: PubMed; (2) Time frame: 2010–2025; (3) Search terms: neuroscience and near-infrared brain functional imaging; Search strategy: (“neuroscience”[MeSH Terms]) AND (“functional near-infrared spectroscopy”[All Fields] OR “fNIRS”[All Fields]) AND (“2010/01/01”[PDAT] : “2025/12/31”[PDAT]); (4) Inclusion criteria: English-language articles and studies on basic and clinical research of neurological disorders (organic diseases); and (5) Exclusion criteria: Non-original research (reviews, meta-analyses, systematic reviews, commentaries, letters) and articles related to mental health and psychology.

## Basic Principles, Key Characteristics, and Application Scenarios of Functional Near-Infrared Spectroscopy

### Development history and basic principles of functional near-infrared spectroscopy

**Figure 2:**
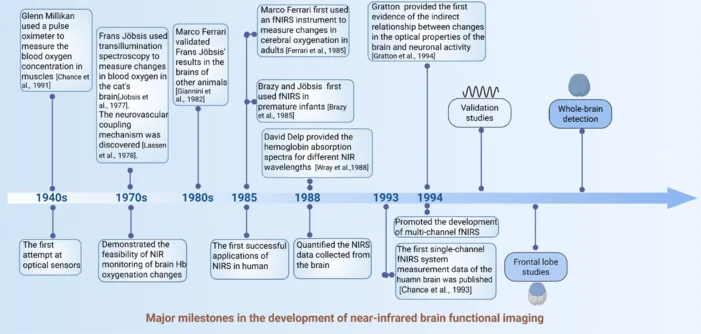
Chronological chart of major milestones in the development of near-infrared brain functional imaging.

As early as the 1940s, Glenn Millikan used a pulse oximeter to measure blood oxygen concentration in muscle, marking the first attempt at optical sensors (Chance, 1991). In the 1970s, Frans Jöbsis employed transillumination spectroscopy to measure changes in blood oxygen levels in the brains of cats, demonstrating the feasibility of NIR monitoring of hemoglobin oxygenation changes in the brain (Jöbsis, 1977). Jöbsis is also considered the founder of *in vivo* near-infrared spectroscopy. The mechanism of near-infrared spectroscopy was discovered by Lassen et al. (1978). Giannini et al. (1982) validated Jöbsis’s results in the brains of other animals (Marco Ferrari’s team). Ferrari et al. (1985) were the first to use an fNIRS instrument to measure changes in cerebral oxygenation in adults. During the same period, Brazy et al. (1985) also used fNIRS to measure changes in cerebral oxygenation in premature infants. These experiments represented the first successful applications of fNIRS in human patients. Later, in 1988, research by David Delp’s team provided hemoglobin absorption spectra for different NIR wavelengths, aiding in the quantification of fNIRS data collected from the brain (Wray et al., 1988). It was not until 1993 that the first single-channel fNIRS system measurement data from the human brain were published (Chance et al., 1993). Gratton and his colleagues provided the first evidence of the indirect relationship between changes in the optical properties of the brain and neuronal activity (Gratton et al., 1994). These efforts promoted the development of multichannel fNIRS. Over nearly 20 years, fNIRS has evolved from first-generation technology (mainly validation studies, which are not suitable for medical applications) and second-generation technology (limited to frontal lobe studies) to third-generation technology (capable of whole-brain detection) (**Figure 2**; Xu et al., 2005; Tian et al., 2009; Klein et al., 2024).

fNIRS is a noninvasive neuroimaging technique based on optical principles, primarily exploiting the differential absorption characteristics of oxyhemoglobin (HbO_2_) and deoxyhemoglobin (HbR) in brain tissue for NIR light in the 600–900 nm range. This enables the real-time and direct detection of hemodynamic activity in the cerebral cortex. By observing such hemodynamic changes, neural activity can be inferred through near-infrared spectroscopy (Almajidy et al., 2020). fNIRS takes advantage of the strong penetrating ability of NIR light in biological tissue (Wyatt et al., 1986). Light propagation depends on the wavelength of the light and the optical properties of the medium, including the reflection, scattering, and absorption of the incident light.

**Figure 3:**
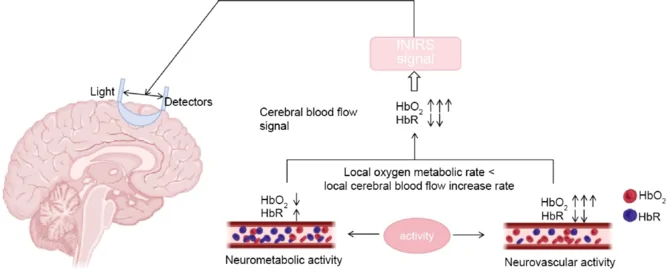
Schematic diagram of fNIRS. fNIRS: Functional near-infrared brain imaging; HbO_2_: oxyhemoglobin; HbR: deoxyhemoglobin.

Reflection depends on the angle of incidence between the light and tissue, as well as the optical density of the material. Absorption depends on the chemical composition of the medium, whereas scattering is influenced by multiple parameters, such as wavelength and particle density (Jobsis, 1977; Klein et al., 2024; Zhang et al., 2024d). Blood absorbance depends on the concentrations of HbO₂, HbR, total hemoglobin, and cytochrome C oxidase, and the concentrations of these components vary with time and metabolic activity (Livecchi et al., 2024; Pellicer et al., 2011). A light source emits NIR light (typically at two or more wavelengths, such as 690 nm and 830 nm) into targeted brain tissue; the light propagates in a banana-shaped scattering path, and a detector collects the light that is scattered back from the brain tissue. NIR can detect cortical regions located 20–30 mm beneath the skull (Song et al., 2019). Based on the correlation between light attenuation and changes in the concentration of tissue chromophores, and according to the modified Beer–Lambert law, fNIRS can be used to quantify variations in the concentrations of HbO_2_ and HbR within brain tissue (Bunce et al., 2006). Changes in HbO_2_ and HbR concentrations are direct readouts of brain function dynamics. When a cortical region is activated, local oxygen metabolism and cerebral blood flow rates are rapidly altered. Initially, neurons consume oxygen to generate energy, causing HbO_2_ levels to decrease and HbR levels to increase (Liao et al., 2013). To meet this heightened metabolic demand, cerebral blood flow surges in the activated area, often outpacing oxygen consumption. The net effect is an oversupply of oxygenated blood; thus, HbO_2_ increases and HbR decreases in the active cortical region (Scholkmann et al., 2014). This hemodynamic signature is precisely what fNIRS detects and quantifies (**Figure 3**).

### Key characteristics and comparisons with other functional near-infrared spectroscopy technologies

Key fNIRS light parameters—such as the light source, wavelength, detector, spatial arrangement of source–detector pairs, and the technical type of source modulation—are essential for enhancing the reliability of fNIRS (Biallas et al., 2012). fNIRS is typically divided into three categories based on the light-source modulation scheme: continuous-wave, frequency-domain, and time-domain. Continuous-wave technology emits NIR light of constant intensity; the detector measures the attenuation produced by absorption and scattering in brain tissue. Because the path length cannot be determined directly, continuous-wave systems cannot quantify absolute concentrations of HbO_2_ and HbR; they assume a fixed path length and therefore report only relative concentration changes (Ferrari et al., 2012). Frequency-domain technology emits intensity-modulated light (typically 100–1000 MHz). As the light propagates through tissue, both its amplitude is attenuated and its phase is shifted. The phase shift allows for the estimation of the effective path length, enabling the calculation of absolute HbO_2_ and HbR concentrations (Stute et al., 2025). Time-domain technology emits ultrashort (picosecond) pulses, and a detector records the temporal distribution of photon arrival times. By analyzing early and late-arriving photons, time-domain systems can separately estimate absorption and reduced-scattering coefficients, yielding the most accurate absolute HbO_2_ and HbR values. Time-domain technology provides the highest precision but is technically the most demanding and expensive (Muehlemann et al., 2011). However, in functional brain imaging, the priority is often to cover a larger cortical area and generate activation maps that pinpoint where activity occurs; absolute quantification is less critical than robust statistical detection of changes (Ferrari et al., 2012). Continuous-wave systems are relatively simple and can still effectively track changes in HbO_2_ and HbR (albeit relatively), making them adequate for most applications. Their key components can be very small, facilitating the development of truly wearable devices. Consequently, continuous-wave technology is the most widely implemented fNIRS approach, and the remaining discussion in this paper will focus on continuous-wave-based fNIRS parameters.

Regarding wavelength, fNIRS imaging typically employs two or a few discrete wavelengths to achieve a higher signal-to-noise ratio (Kocsis et al., 2006). In broadband fNIRS, a continuous spectral band is required to obtain the continuous absorption spectrum of diffuse reflectance (Diop et al., 2009). The NIR region is divided into two windows: NIR-I (650–1000 nm) and NIR-II (1000–1700 nm). Because water absorption increases sharply beyond 900 nm and overtakes that of HbR and HbO_2_, virtually all current fNIRS imagers operate within the NIR-I band (Chen et al., 2020). Driven by continuous advances in fluorescent probes, NIR-II-window fluorescence detection has emerged as a novel *in vivo* imaging modality that combines high spatial resolution (approximately 30 μm) and temporal resolution (< 200 ms per frame) with millimeter-scale tissue penetration. Rare-earth-based luminescent probes exhibit both upconversion and downconversion emissions spanning 300–3000 nm and have become pivotal in *in vivo* NIR-II bioimaging, including vascular and molecular imaging. It is hoped that forthcoming clinical translations will overcome the current limitation of insufficient brain penetration depth by NIR-I (Zhong et al., 2020). Researchers have constructed NIR-II optical microscopes that enable noninvasive, volumetric 3D multiplexed molecular imaging of the CT26 tumor microenvironment in mice, while providing longitudinal tracking of CD4, CD8, and OX40 expression at single-cell resolution after immunotherapy. In follow-up work, the same group trained artificial neural networks to transform NIR-I images into NIR-II-like images, further improving the precision of tumor margin delineation (Ma et al., 2021, 2022; Wang et al., 2021a, b, 2023a). As this review focuses on the clinical applications of fNIRS in neurological disorders, the imaging systems discussed herein predominantly employ NIR within the NIR-I window, using changes in HbR and HbO_2_ concentrations to index aberrant brain activity and functional connectivity. The wavelength of approximately 805 nm is known as the isosbestic point, where the absorption coefficients of HbO_2_ and HbR are equal. For continuous-wave-based fNIRS systems, two wavelengths are usually selected: one on each side of this point, i.e., a shorter wavelength at which HbR absorbs more strongly than HbO_2_ (e.g., 690 nm) and a longer wavelength at which HbO_2_ absorbs more strongly than HbR (e.g., 830 nm). When three or more wavelengths are employed, the third is typically chosen close to the isosbestic point (Wyser et al., 2017).

The light sources most commonly used in functional near-infrared spectroscopy (fNIRS) are laser diodes and light-emitting diodes (Scholkmann et al., 2014). Lasers offer superior stability and a narrow bandwidth (high spectral purity), allowing them to deliver higher optical power. However, they are more expensive and sensitive to ambient temperature, which is why they are typically used in large, stationary fNIRS systems. In contrast, light-emitting diodes are compact, inexpensive, and easily integrated into wearable fNIRS devices. However, they have a broader spectral bandwidth, lower optical power, and poorer stability (Almajidy et al., 2020).

There are generally three types of detectors: photodiodes, avalanche photodiodes, and photomultiplier tubes. Photodiodes are small, inexpensive, and resistant to environmental interference, but they do not provide internal signal amplification, resulting in low sensitivity. Avalanche photodiodes offer moderate gains but are temperature-sensitive, often requiring temperature stabilization components. Photomultiplier tubes provide the highest gain and can resolve single photons; however, they are highly sensitive to external conditions, such as power supply fluctuations, magnetic fields, and ambient light, and are the most expensive of the three types of detectors (Scholkmann et al., 2014). The most commonly used detectors in fNIRS are photodiodes and avalanche photodiodes (Scholkmann et al., 2014).

To achieve high image resolution and sensitivity, accurately estimate chromophore concentrations, and effectively suppress contributions from superficial tissue layers as well as systemic physiological fluctuations, careful optimization of the source–detector geometry is essential (Scholkmann et al., 2014). The source–detector geometry directly determines the penetration depth of fNIRS: the greater the source–detector separation, the deeper the light travels into the brain, although the detected intensity decreases exponentially. At a source–detector distance of approximately 3 cm (2.5 cm for infants), cortical hemodynamics can be measured. Separations of 1 cm or less form “short-separation” channels that predominantly capture superficial scalp signals, which are essentially physiological noise. Many current systems therefore include such short channels; their signals are later regressed out of the long-separation data to improve the specificity of cortical recordings (Collins-Jones et al., 2021). We have consolidated the key fNIRS device parameters for neurological applications in **Additional Table 1** to serve as a quick reference for clinicians.

**Additional Table 1:** Characteristics of fNIRS devices for neurological disease applications

	Light-source wavelength	Device type	Light type	Emitter-detector distance	Sampling rate
Stroke	650-900 nm, mainly 730, 760, 850 nm	Continuous-wave	LDs	3 cm	3.91-20 Hz, mainly 10, 11 Hz
PD	760, 850 nm	Continuous-wave	LED	3, 3.5, 4 cm	7.81-50 Hz, mainly 10 Hz
AD	760, 850 nm, 695-850 nm, mainly 695, 850 nm	NA	LED	3-4 cm, mainly 3 cm	3.91-19 Hz
ALS	760, 850 nm	NA	NA	3 cm	7.81-15.6 Hz
Epilepsy	690-850 nm, mainly 690,830 nm	NA	NA	2.5-5.0 cm, mainly 3-5 cm	19.5-20 Hz
SCI	690-860 nm	Continuous-wave	NA	3.0-3.5 cm	10-25 Hz
TBI	730, 760, 850 nm	Continuous-wave	LED	3.0-3.5 cm	2-3.47 Hz
Migraine	730, 760, 850 nm	Continuous-wave	LED	2.5 cm	7.81 Hz
Dystonia	760, 850 nm	Continuous-wave	LED	3 cm	7.81 Hz
DAVFs	760, 850 nm	NA	NA	NA	7.825 Hz
DMD	735, 810, 850 nm	Continuous-wave	LED	3 cm	60 Hz
MS	730, 760, 850 nm	Continuous-wave	LED	2.5-4.0 cm	2, 7.81 Hz

The commonly used fNIRS parameters in neurological diseases provide a data reference for clinicians and rehabilitation therapists. AD: Alzheimer's disease; ALS: amyotrophic lateral sclerosis; DAVFs: dural arteriovenous fistulas; DMD: Duchenne muscular dystrophy; fNIRS: functional near-infrared spectroscopy; LDs: laser diodes; LED: light-emitting diodes; MS: multiple sclerosis; NA: not applicable; PD: Parkinson's disease; SCI: spinal cord injury; TBI: traumatic brain injury.

fNIRS is a noninvasive brain imaging modality that offers unique advantages in clinical settings. In functional brain imaging, fNIRS is commonly used alongside other techniques such as fMRI, PET, EEG, and MEG. fMRI measures hemodynamic changes following enhanced neural activity, and its application functions similarly to that of fNIRS. The key difference is that fMRI works by measuring the correlation between spontaneous low-frequency fluctuations in different voxels and blood oxygen level-dependent (BOLD) signals (Logothetis, 2008; Baracchini et al., 2023; Huang et al., 2023). Previous studies have shown that the hemoglobin signal obtained via fNIRS is strongly correlated with BOLD signals obtained via fMRI, with consistent localization of functional brain regions (Cui et al., 2011; Sato et al., 2013; Struckmann et al., 2022). Although the spatial resolution and depth of penetration of fNIRS are inferior to those of fMRI, fNIRS also targets hemodynamic changes. fNIRS operates at sampling rates of 10–100 Hz (i.e., one acquisition every 0.1–0.01 seconds), whereas a whole-brain fMRI volume requires 1–2 seconds. This high temporal resolution allows fNIRS to capture rapid hemodynamic transients more accurately. Moreover, fMRI imposes inherent exclusion criteria, making children, elderly individuals, pregnant women, or those with metallic implants often unsuitable for scanning. In contrast, fNIRS has no such contraindications, and all populations tolerate low-energy near-infrared light well (Li et al., 2019). PET uses radioactive tracers to functionally image metabolic processes, blood flow, regional chemical composition, and/or chemical absorption. PET can be combined with computed tomography or MRI structural imaging to improve detection accuracy through attenuation correction (Liu et al., 2023a, b, c). In neurological disorders, blood–brain-barrier-penetrating tracers that selectively quantify target–ligand interactions are now available. These agents have enhanced our understanding of the pathological mechanisms underlying Alzheimer’s disease (AD), PD, epilepsy, and brain tumors, providing powerful tools for discovering new therapeutic targets (Liu et al., 2023a, b, c). While PET technology offers high spatial resolution and sensitivity, its high cost and use of radioactivity are notable drawbacks (van der Meulen et al., 2021).

EEG, another widely used technique, records the minute electrical potentials generated by large-scale neuronal synchrony, directly reflecting neuronal firing and thus providing submillisecond temporal resolution. This outperforms the comparatively slow hemodynamic response monitored by fNIRS (Coquelet et al., 2020). Furthermore, because hemodynamics is a metabolic consequence of neural activity, fNIRS provides only an indirect index of neuronal events. However, volume conduction severely limits the spatial accuracy of EEG, whereas fNIRS, with its sensitivity confined to the cortical region underlying the optode array, results in far better spatial localization. Additionally, fNIRS is markedly more robust to motion artifacts than EEG, making it suitable for ecologically valid paradigms that require overt movement, such as assessments of motor function in PD or stroke (Pernet et al., 2020; Hallett et al., 2021). MEG also detects electromagnetic brain activity—the tiny magnetic fields produced by synchronous neuronal currents—and, unlike EEG, can sample deep sources. In other respects, MEG shares the merits and limitations of EEG. Invasive approaches such as electrocorticography or local field potentials combine excellent spatial and temporal resolution but require craniotomy and carry surgical risks (Anzellotti et al., 2016). With technological advances, high-density EEG arrays with more than 64 channels (128, 256, or even 320 electrodes) placed on the scalp have gained widespread clinical use. A previous study has shown that such high-density recordings can yield resting-state functional connectivity maps similar to those obtained with MEG (Coquelet et al., 2020). Consequently, fNIRS occupies a distinctive niche: it is noninvasive, offers better temporal resolution than fMRI, better spatial resolution than EEG and MEG, and is tolerant of motion, making it applicable to a broader range of clinical populations and experimental contexts (**Additional Table 2**).

**Additional Table 2:** Comparison of fNIRS and fMRI imaging techniques

	Detection parameters	Spatial resolution	Time resolution	Anti-motion interference	Anti-electromagnetic interference	Movability	Prices	Novelty
fMRI	Blood flow	+++	+	+	NA	+	+++	+
PET	Glucose metabolism	+++	+	+	NA	+	++++	+
EEG	Electrical activity	+	+++	+	+	++	+	+
MEG	Magnetic field signal	++	+++	+	NA	+	++	++
fNIRS	Blood flow	++	++	+++	+++	+++	++	+++

This table compares fNIRS with other brain functional imaging techniques, highlighting the advantages of fNIRS. EEG: Electroencephalography; fMRI: functional magnetic resonance imaging; fNIRS: functional near-infrared spectroscopy; MEG: magnetoencephalography; PET: positron emission tomography; +: The symbol "+" denotes degree: the greater the number of "+" signs, the more pronounced the corresponding characteristic. The relative number of "+" signs indicates the strengths and weaknesses of the key features of each imaging technique.

### Functional near-infrared spectroscopy application scenarios

Owing to its distinctive advantages—noninvasiveness, resistance to motion artifacts, and portability/wearability—fNIRS is now used across a wide range of fields. As a brain-imaging modality, fNIRS enables the mechanistic dissection of brain functional states and cognitive neural activity. Its high ecological validity overcomes the traditional limitation of studying attention, cognition, language, and other processes exclusively in laboratory settings, offering a tool for investigating brain activity in naturalistic environments. For example, researchers have used fNIRS to examine interpersonal neural synchrony during real classroom interactions between teachers and students and to explore multiplayer decision-making in card games (Li et al., 2024a, b; Zhang et al., 2024a, b, c). Due to its child-friendly characteristics, fNIRS is also a powerful tool for studying early human development and functional brain mapping (Zhang et al., 2021). In human–machine interactions, fNIRS has proven advantageous for monitoring mental fatigue in pilots and drivers and for investigating consumer choices during free movement (Deng et al., 2025; Zhao et al., 2025). Additionally, fNIRS can be used to simultaneously record the brain activity of both speakers and listeners, allowing for interbrain analyses to examine dyadic speech communication in noisy environments (Li et al., 2021). It is also employed to investigate how interpersonal neural coordination underpins social cooperation (Zhou et al., 2022). In the context of public safety, fNIRS can be integrated into handheld spectrometers to enhance security screening in large-scale social environments such as subways, airports, and train stations (Crocombe, 2018). For everyday wellness, wireless fNIRS headsets paired with smartphone apps now allow for the simultaneous tracking of vital signs and brain function in elderly individuals (Phillips et al., 2023). The screening, diagnosis, and evaluation of functional brain disorders are increasingly among the primary application scenarios for fNIRS. It is already used in psychiatry, rehabilitation, geriatrics, neurology, and pediatrics to monitor region-specific brain activity and to aid in gait recovery for stroke and PD patients (Abtahi et al., 2020; Saway et al., 2024). Moreover, when combined with other modalities, fNIRS can help identify biomarkers for AD and schizophrenia (Diao et al., 2024; Mei et al., 2024) and provide continuous cerebral monitoring in critical care units, guiding ventilator settings (Mathur et al., 2024). Finally, in the field of brain–computer interfaces (BCIs), fNIRS is being combined with EEG for simultaneous signal acquisition and decoding, with applications already reported in stroke and ALS patients (Rea et al., 2014; Hosni et al., 2020). The following sections focus on the clinical applications of fNIRS in neurological disorders.

## Application of Functional Near-Infrared Spectroscopy in Stroke

### Application of functional near-infrared spectroscopy in stroke rehabilitation

Epidemiological data indicate that stroke remains a prevalent condition that jeopardizes the safety and quality of life for patients worldwide. According to the China Stroke Surveillance Annual Report 2021 Edition, 17.8 million adults in China have experienced a stroke, with 3.4 million new strokes occurring in 2020 and an additional 2.3 million deaths attributed to stroke. Approximately 12.5% of stroke survivors live with disabilities and require long-term rehabilitation to regain their ability to live independently (Tu et al., 2023; Hilkens et al., 2024; Feigin et al., 2025). Impaired structural connections in the motor cortex and disrupted subcortical network connections contribute to these disabilities in stroke patients (Tu et al., 2023; Hakon et al., 2024). Notably, brain dysfunction following a stroke arises not only from structural disconnection around the lesion but also from the disruption of functional networks across brain regions and between the cerebral hemispheres (Shen et al., 2023, 2025). Restoring function and FC within both hemispheres is crucial for motor rehabilitation, a process known as cortical reorganization (Grefkes et al., 2011; Sparling et al., 2024). Cortical reorganization can be understood as the process through which, influenced by various rehabilitation training methods (behavioral or neuromodulatory), new connections are established within the functional networks of the brain, or corresponding neuronal activities are activated or inhibited to compensate for impaired motor functions (Paul et al., 2023; Tscherpel et al., 2024). Research has shown that when a stroke lesion is small, restoring functional mapping and reorganization in adjacent brain regions is often sufficient. However, when the lesion is large, functional mapping and reorganization may require the recruitment of more distant and contralateral brain regions, likely due to more severe structural damage around the lesion (Hakon et al., 2024; Shen et al., 2025). Given this understanding, accurately characterizing cortical reorganization is essential for improving rehabilitation outcomes for stroke patients. Previously, fMRI was commonly used to characterize cortical reorganization, typically measuring resting-state FC before and after rehabilitation interventions (Guo et al., 2023; Liu et al., 2024). However, such measurements may not adequately reflect the true activity state of the brain, particularly during rehabilitation training. Time series-based imaging combined with modeling analysis can help determine effective connectivity and provide feedback on a more realistic state of brain function. However, each analytical model has its limitations, and no single model can be universally applied across all experimental conditions and datasets (Zhang et al., 2024a, b, c). In this context, fNIRS, due to its convenience, can be employed to measure brain activity synchronously during rehabilitation training and can also be combined with neuromodulation techniques to provide real-time feedback on the effects of interventions. Therefore, we believe that fNIRS can become a valuable tool for characterizing cortical reorganization during rehabilitation and can aid in assessing rehabilitation efficacy. Some experimental results have already supported our hypothesis. For instance, in stroke patients, increased brain activity in the sensorimotor cortex (SMC) has been observed during upper limb training, and enhanced FC between the prefrontal cortex (PFC) and primary motor cortex (M1) has been noted during task-oriented training (Lim et al., 2019; Huo et al., 2023; Martio et al., 2023; Peng et al., 2023). In the following paragraphs, we will detail the application of fNIRS in post-stroke rehabilitation training (**Additional Figure 1**). Finally, based on existing application outcomes and future development trends, this review will explore more scientific clinical translation ideas and methods for fNIRS.

Even in the modern era, where numerous neuromodulation technologies and even brain–computer interfaces have emerged, exercise rehabilitation has always been an essential component of poststroke rehabilitation, especially in widely economically underdeveloped areas (Bei et al., 2023; Ospel et al., 2024). Previously, the degree of motor recovery was assessed mainly using classical motor function scales such as the Brunnstrom Motor Recovery 6-Grade Staging Scale, the Fugl–Meyer Motor Rating Scale, and the Modified Ashworth Spasticity Rating Scale. However, these scores are rather subjective and do not directly reflect the recovery of motor cortex functions (Li et al., 2020a, b, 2022; Mani et al., 2013). The accurate determination of the controllability function of the motor cortex in the brain is crucial for the rehabilitation of stroke patients and is a key part of cortical reorganization. As an instrument that can objectively measure brain function, fNIRS, when used in conjunction with scores before and after rehabilitation training, can reflect not only improvements in motor function but also changes in brain function, which is also evidence of cortical reorganization. A significant correlation between fNIRS-measured brain activity and scale scores has been observed. Impaired brain activity and decreased motor scores improve significantly after stroke patients undergo rehabilitation (AlQahtani et al., 2024; Bu et al., 2023). Li et al. (2020a, b) reported that the activation of the ipsilateral primary motor cortex (M1) is positively correlated with motor rehabilitation, while the FC between the ipsilateral supplementary motor cortex (SMA) and M1 is negatively correlated with motor rehabilitation. Additionally, the increase in motor scores following rehabilitation is accompanied by an increase in ipsilateral M1 activation and enhanced FC between the bilateral M1 (Li et al., 2020a, b). The results reported by Liang et al. (2022) revealed that event-related desynchronization, HbO_2_, and the phase synchronization index detected by fNIRS and EEG are predictive biomarkers of Berg Balance Scale scores when stroke patients perform ankle dorsal zone exercises. Therefore, fNIRS can be utilized as a measure of cortical reorganization to predict and assess the effectiveness of exercise rehabilitation in stroke patients.

The neuromodulation technique is effective for improving motor function in stroke patients. Some common neuromodulation methods include transcranial direct current stimulation (tDCS), median nerve electrical stimulation, fastigial nucleus stimulation, focal muscle vibration, robot-assisted upper limb training, and transcutaneous auricular vagus nerve stimulation (Keser et al., 2023; AlQahtani et al., 2024; Saway et al., 2024). A key issue that currently exists is the selection of neuromodulation targets, which is inconsistent. As the main site of motor output, M1 is mostly selected as the target site (Lefaucheur et al., 2020; Badran et al., 2023). In addition, the SMA is also an important target for neuromodulation. The SMA is functionally connected to M1 and is involved in cortical-to-spinal cord projections (Diao et al., 2017; Windhorst et al., 2023). Other studies have selected corresponding modulation targets based on the results of previous basic experiments or clinical experience (Baker et al., 2023; Liao et al., 2024a, b). Admittedly, these trials have also reported statistically significant effects of neuromodulation. However, we must also recognize that an increasing body of evidence suggests that the effects of neuromodulation techniques (such as transcranial magnetic stimulation) are target dependent. Precisely, targeting specific areas may improve clinical outcomes for any given symptom; that is, stimulating different targets may improve different symptoms (Bai et al., 2023; Siddiqi et al., 2024). Considering the heterogeneity of stroke lesion location, size, and other factors, the development of targeted neural interventions is expected in this era of personalized precision medicine. By modulating the SMA and its related networks using neuromodulation techniques, Mihara et al. (2021) used fNIRS to provide real-time feedback on SMA neural activity during motor imagery, which enhanced post-stroke gait and balance recovery. These experimental results suggest that the application of fNIRS can help researchers evaluate whether the modulation sites selected by the neuromodulation technique are appropriate and effective (Kim et al., 2023; Cai et al., 2024). Furthermore, considering differences such as head shape and the distance from the scalp to the target, shifting targets based on patient responses to identify the optimal target can achieve the goal of individualized modulation. In addition to assisting in the selection of modulation targets for neuromodulation techniques, fNIRS can also be used to monitor neuromodulation efficacy. Previous studies have shown that many neuromodulation techniques can improve activity and functional connectivity in brain regions associated with motor function, including the PFC, occipital cortex (OC), somatosensory association cortex, brainstem, superior frontal cortex, and Broca’s area (Huo et al., 2019; Siddiqi et al., 2024). We summarized the current research on the use of fNIRS to detect changes in brain activity and functional connectivity after neuromodulation treatment (**Additional Table 3**). These studies suggest that the motor cortex (including M1, premotor cortex [PMC], and SMA) can exhibit robust plasticity to facilitate motor recovery in stroke patients.

**Additional Table 3:** Application of fNIRS in neuromodulation rehabilitation of stroke

Study	Neuromodulation methods	Research population	Stimulus area	Experimental design	Primary endpoint events	Brain activity and functional connectivity results
Kim et al., 2023	HD-tDCS	Chronic stroke patients	C3 or C4 (based on the 10-20 system)	A sham-controlled crossover study design: (a) real HD-tDCS and (b) with sham HD-tDCS; Both groups performed the sequential finger tapping task	Motor performance as shown in response time during the SFTT	Real HD-tDCS increased brain activity of ipsilesional M1 and FC between ipsilesional M1 and PMC
Huo et al., 2019	MNES	Right hemiplegia stroke patients	Right wrist	Self-controlled studies: resting and MNES-stimulated states	Cerebral oxygenation signals in the LPFC/RPFC, LM1/RM1, and LOC/ROC	MNES increased the FC from LPFC and RPFC to LOC, LM1 and LOC to RPFC
Li et al., 2024a	Dual-tDCS	Subacute stroke patients	PSC	A randomized, double-blinded, sham-controlled study: dual-tDCS group and sham-tDCS group	FMA-UE scores before and after treatment	Dual-tDCS increased the brain activity of left PFC and left SMA. Dual-tDCS increased the FC between left SAC and right PMC-SMA.
Ma et al., 2022	Electrical stimulation of the cerebellar fastigial nucleus	Stroke patients	Cerebellar fastigial nucleus	A case-control study: fNS electrical stimulation was applied to stroke patients and healthy controls	Brain activity of brain cortex	fNS electrical stimulation increased the brain activity of bilateral PFC and bilateral M1.
Shen et al., 2023	FMV	Right hemiplegia stroke patients	Forearm flexor muscles	Self-controlled studies: before and after FMV	Brain activity of bilateral PfC, SMA, and OC	FMV increased the brain activity of bilateral PFC, bilateral SMA and left OC.
Xie et al., 2022	Robot-assisted task-oriented UL motor training	Adult stroke patients with hemiplegic motor impairment	Upper limb	Self-controlled studies: resting and robot-assisted task-oriented UL motor training	Brain activity of bilateral PfC, DLPFC, SFC, PMC, M1, S1, and OC	UL motor training increased brain activity of ipsilesional SFC in the mild group and bilateral SFC in the moderate group.
Wang et al., 2023a	TaVNS	Stroke patients presenting with either left or right hemiparesis	Left ear	Self-controlled studies: before and after taVNS	Brain activity of brain cortex	TaVNS increased the brain activity of left PMC, SMA, and Broca's area in patients exhibiting left-side hemiplegia. taVNS increased the brain activity of left S1 in patients exhibiting right-side hemiplegia.
Mihara et al., 2021	fNIRS-NFB	Patients with subcortical stroke-induced mild to moderate gait disturbance more than 12 weeks from onset	SMA	A randomized controlled trial: fNIRS-NFB group or placebo group	Timed up and go test outcomes	fNIRS-NFB increased brain activity of SMA and FC between SMA and PMC.

This table presents the status of fNIRS applications in the stroke field and emphasizes the value of fNIRS in cortical reorganization and neuroregeneration during rehabilitation therapy. Dual-tDCS: dual transcranial direct current stimulation; DLPFC: dorsolateral PFC; FC: functional connectivity; FMV: focal muscle vibration; fNIRS: functional near-infrared spectroscopy; fNIRS-NFB: fNIRS mediated neurofeedback; fNS: fastigial nucleus stimulation; HD-tDCS: high-definition transcranial direct current stimulation; LOC: left occipital cortex; LPFC: left prefrontal cortex; Ml : primary motor cortex; MNES: median nerve electrical stimulation; OC: occipital cortex; DLPFC: dorsolateral prefrontal cortex; SFC: superior frontal cortex; PFC: prefrontal cortex; PMC: premotor cortex; PSC: primary somatosensory cortex; RPFC: right prefrontal cortex; S1: sensory cortex; SAC: somatosensory association cortex; SFC: superior frontal cortex; SMA: supplementary motor cortex; taVNS: transcutaneous auricular vagus nerve stimulation; UL: upper limb.

Brain–computer interface (BCI) is a technique that utilizes neurophysiological or brain metabolic activity to drive machines with the goal of modulating brain activity, reorganizing cortical function, and promoting motor rehabilitation (Liu et al., 2023a, b, c; Brunner et al., 2024). Advancements in artificial intelligence and machine learning have enhanced the adaptability and accuracy of BCIs. Interdisciplinary collaboration among neuroscience, engineering, and clinical fields has fueled the innovation and practical implementation of BCI technologies. BCI technologies are categorized into three types based on invasiveness: invasive, noninvasive, and seminvasive. This review primarily discusses the clinical application progress of fNIRS in noninvasive BCIs (Wang et al., 2024a, b). A central issue in the application of BCI technology is the decoding of brain activity signals and their conversion into commands that can be understood by external devices. EEG can capture the activity of neurons in the cerebral cortex in real-time and is characterized by high temporal resolution, making it the most commonly used method for signal recording in noninvasive BCIs. However, EEG also has limitations, such as difficulty in exploring signals from deep brain regions and relatively low spatial resolution (Prasad et al., 2022; Huang et al., 2024). Compared with EEG, fNIRS is less affected by electrical artifacts and can also measure cortical neural activity (Zhang et al., 2024a, b, c). A literature review revealed that fNIRS has been used to assist in lower limb motor rehabilitation with BCIs. One study demonstrated that fNIRS could measure hemodynamic changes in the brain during right and left hip movements in stroke patients (Rea et al., 2014). Experiments by Liu et al. (2022) confirmed that BCIs significantly increase functional connectivity from the ipsilateral M1 to the frontal lobe, and that this change was instantly captured by fNIRS. These results illustrate attempts to apply fNIRS in BCIs. However, current studies lack results that highlight the advantages of fNIRS over EEG (Patel et al., 2020; Zhang et al., 2024a, b, c). Research has also explored integrating feedback modes from multiple sensory pathways, including visual, auditory, and tactile stimuli, using combined EEG and fNIRS detection to evaluate the rehabilitation effects of BCI applications in stroke patients. Wang et al. (2022a, b) designed a multimodal neurofeedback training system (BCI–NFT–functional electrical stimulation) for the rehabilitation of stroke patients, utilizing combined EEG and fNIRS monitoring. After 4 weeks of training, the cerebral oxygen response and motor scores of stroke patients improved significantly, indicating that the application of the NFT–BCI in stroke rehabilitation holds considerable promise. The real-time monitoring feature of fNIRS enables it to establish a closed-loop neurofeedback training pathway with BCIs, allowing subjects to modulate motor intention based on brain activity at any time. Corresponding stimuli (such as functional electrical stimulation) are then applied based on the real-time feedback results from the online model to maximize neural remodeling. Currently, the application of fNIRS in BCIs remains relatively limited. Given the complexity of decoding the input and output signals of BCIs, combining fNIRS with EEG or other complementary technologies may be a more practical and feasible approach.

In high-functioning stroke patients, decreased hand coordination and fine motor dexterity are major disorders affecting their daily lives (Patel et al., 2020). Most previous studies have focused on gross motor rehabilitation, while the mechanisms of fine motor coordination and rehabilitation training have received less attention (Fisher et al., 2022; Chen et al., 2024e). Chen et al. (2024e) used fNIRS to measure cortical responses during the execution of fine motor control and coordination activities in high-functioning stroke patients. The results revealed significant activation of the bilateral SMC when patients performed a bimanual coordination task. Additionally, patients who demonstrated better performance in the bimanual task exhibited more efficient cortical activation. Therefore, bilateral arm training is crucial for the rehabilitation of high-functioning stroke patients. These findings suggest that understanding the neural mechanisms underlying fine motor and coordinated activities, along with the combination of fNIRS and motor paradigms, could aid researchers in developing more scientific and individualized rehabilitation programs for stroke patients.

As described above, fNIRS can be used for the effective assessment of exercise rehabilitation, selection of neuromodulation targets, and evaluation of the efficacy of neuromodulation techniques, can be combined with BCIs to assist in limb motor rehabilitation, and can be used in studies of fine motor rehabilitation mechanisms (Patel et al., 2020; Fisher et al., 2022). However, there are still several limitations with regard to the current application of fNIRS. For example, in motor rehabilitation, fNIRS is only used in combination with motor function scores to assess the effectiveness of rehabilitation and achieve objective and scientific rehabilitation outcomes. With regard to neuromodulation, fNIRS is mainly used to evaluate the effects of modulation and the corresponding improvements in brain function. There is currently only one study reporting the use of fNIRS for real-time feedback on modulation targets. In the future, with the help of artificial intelligence, machine learning, and big data models, fNIRS should be promoted to play a more in-depth and precise role in stroke rehabilitation. For example, stroke patients can wear portable fNIRS devices during motor training. Training intensity and methods can be adjusted on the basis of the neural activity of the motor cortex and other related brain regions to find the most suitable treatment plan for each patient. fNIRS can be used to measure brain activity simultaneously with neuromodulation. The target can be determined on the basis of the neural activity response, and microshifts can be made according to patient response to achieve the best effect. To achieve these goals, the first step is to engage in a global collaborative effort to advance the standardization of fNIRS technology, promote data sharing, and establish a big data model for stroke patients using fNIRS. The second step is to use artificial intelligence to identify the unique brain activity patterns of each stroke patient and customize individualized treatment plans; moreover, machine learning algorithms can identify subtle differences on the basis of a big data model to improve the accuracy of treatment plans. The third step in the development of fNIRS applications is facilitating the cooperation of multiple disciplines during the advancement process. For example, neuroscientists provide insights into brain function, biomedical scientists develop sophisticated algorithms, clinicians provide real feedback on patient treatment outcomes, and engineering professionals further optimize the equipment. This is particularly important for stimulating innovation and clinical application.

### Application of functional near-infrared spectroscopy in stroke comorbidities

The prevalence of cognitive dysfunction among ischemic stroke patients, particularly postischemic stroke executive impairment (PISEI), is a significant concern, affecting approximately two-thirds of this population. PISEI not only diminishes the quality of life for these patients but also hinders their ability to reintegrate into the workforce (Wu et al., 2019; Pandian et al., 2020; Ye et al., 2023). Traditional methods for diagnosing PISEI primarily hinge on clinical symptoms and neuropsychological evaluations, highlighting a gap in objective and direct biological markers for this condition (McGovern et al., 2016).

Cognitive–behavioral training is the predominant rehabilitation approach for managing PISEI; however, it is often criticized for being time-consuming, labor-intensive, and slow-acting, with many patients showing poor adherence to the regimen (Quinn et al., 2018). Research suggests that the bilateral fronto-cingulate-parietal network plays a crucial role in both attention and executive function, yet there is a noticeable lack of imaging studies exploring the functional impairments of these neural networks in relation to PISEI (Liu et al., 2023a, b, c; Karnadipa et al., 2025). Recent studies utilizing fNIRS have shed light on the neural activity associated with PISEI. For instance, Liu et al. (2023a, b, c) demonstrated that ischemic stroke patients with PISEI exhibited significantly reduced brain activity in regions such as the left dorsolateral prefrontal cortex (DLPFC), PMC, and right SMC when engaged in executive function tasks. Notably, after undergoing transcranial magnetic stimulation (TMS), these patients showed marked improvements in both brain activity and executive function scores (Liu et al., 2023a, b, c). Furthermore, Zou et al. (2023) found that FC within the SMA, DLPFC, and medial PFC was lower in PISEI patients compared with healthy controls. This suggests that fNIRS may not only serve as a diagnostic tool for PISEI but also as a valuable instrument for monitoring rehabilitation progress.

In addition to cognitive impairments, poststroke depression (PSD) affects roughly one-third of stroke survivors, with its underlying mechanisms remaining poorly understood (Guo et al., 2022; Chen et al., 2024d). The development of PSD may be linked to damage in specific brain networks (Boes et al., 2015; Zhou et al., 2024a, b). However, previous investigations have largely concentrated on resting-state brain networks, yielding inconclusive findings regarding the functional alterations contributing to PSD (Peng et al., 2023; Zhou et al., 2024a, b). Notably, while brain networks identified in resting states also appear during task performance, variations in functional connectivity patterns between these states have been observed (Foster et al., 2015). In a recent study, Peng et al. (2023) performed fNIRS to analyze the functional connectivity of stroke patients with and without PSD during both resting and oddball task states. Their findings indicated that the oddball task state correlated more strongly with the brain network properties associated with PSD compared with the resting state. Specifically, the PSD group exhibited diminished capacity for resilience and information transmission within their brain networks during the oddball task, highlighting the need for further exploration into the functional dynamics of brain networks in relation to PSD. Overall, these studies emphasize the potential of fNIRS as a versatile tool in both diagnosing and monitoring cognitive and affective impairments in stroke patients, paving the way for more targeted rehabilitation strategies.

Although PISEI and PSD do not affect the daily living abilities of stroke patients as paralysis does, they can still impact work and learning abilities, resulting in many obstacles in the lives of stroke patients. Researchers pay far less attention to these patients than to motor function recovery. As discussed in the previous two paragraphs, fNIRS can be used to help determine changes in brain function status, but its current applications are relatively simple. In the future, standardized and comprehensive psychobehavioral assessments should be conducted for patients with PISEI and PSD in conjunction with comprehensive and standardized neuroimaging scans to elucidate the underlying neural mechanisms in these patients. In addition, mapping the relationship between behavior and the brain is very important. Univariate and multivariate analyses each have their own advantages and disadvantages. As discussed in Section “Development history and basic principles of functional near-infrared spectroscopy,” addressing these issues requires global collaboration to develop big data models, optimization algorithms assisted by artificial intelligence and machine learning, and multidisciplinary integration and innovation (such as neuroscientists, psychiatrists, neurologists, and rehabilitation specialists).

In summary, fNIRS is currently used mainly in the field of stroke for predicting exercise rehabilitation efficacy and monitoring treatment. In addition, fNIRS can be used in combination with other therapeutic techniques to elucidate the neural mechanisms of rehabilitation and facilitate the application of new technologies. Moreover, the diagnosis and treatment of stroke patients with comorbidities, such as PSD and PISEI, can be aided by fNIRS.

## Application of Functional Near-Infrared Spectroscopy in the Treatment of Neurodegenerative Diseases

### Application of functional near-infrared spectroscopy in Parkinson’s disease

fNIRS is increasingly used in neurological disorders; in PD, it reveals altered cortical activity during gait and treatment (**Additional Figure 2**).

#### Functional near-infrared spectroscopy-assisted probing of the mechanisms involved in gait disturbances in patients with Parkinson’s disease

PD is a common neurodegenerative disease that can be categorized into two subtypes on the basis of symptoms: tremor dominant and postural instability gait disorder. Previous studies have shown that damage to different cortical and subcortical pathways is present in patients with these two subtypes (Jiang et al., 2016; Ben-Shlomo et al., 2024; Morris et al., 2024). PD patients have severe gait disturbances, typically slow starts, short stride lengths, fast walking, and difficulty stopping (called panic gait). Traditionally, the diagnosis of gait disorders has relied primarily on the subjective judgment of neurologists during brief outpatient or inpatient consultations. In addition to pharmacological treatment, physical exercise is a major therapeutic strategy for gait disorders in PD patients. In recent years, with the development of sports medicine, such as motion assessment based on wearable motion sensors combined with algorithmic models, there is not only hope for the objective diagnosis of gait disorders but also the customization of individualized neuromodulation therapies and exercise training prescriptions, with regular adjustments according to recovery progress (Burtscher et al., 2024; van Midden et al., 2024). Despite the increasing availability of these sports medicine methods, investigations into the underlying neural mechanisms are still needed. Studies have shown that impairment of the cortico-basal pathway is a potential mechanism underlying the impaired gait of PD patients (Beretta et al., 2020; Anjum et al., 2024; Bove et al., 2024; Nakamura, 2025). The cortico-basal pathway is a functional subregion known as the motor circuit and is composed of the basal ganglia structures, thalamus, and motor and premotor cortices. This motor circuit includes both direct and indirect pathways. The direct pathway originates in the striatum and projects to the substantia nigra pars reticulata and internal globus pallidus, and the indirect pathway originates in the striatum and projects to the external globus pallidus, which in turn projects to the subthalamic nucleus and then to the substantia nigra pars reticulata and internal globus pallidus. The two pathways intersect and complement each other and are regulated by the motor and premotor cortices, which work together to maintain gait stability (Mirzac et al., 2023; Steiner et al., 2024). Many previous studies have documented the characteristic neural activities of direct and indirect pathways, which are considered markers of PD (Darcy et al., 2022; Lofredi et al., 2023). Those studies have focused primarily on abnormalities in the basal ganglia, with insufficient attention given to impaired cortical function, and most are preclinical studies in nonhuman primates. Some researchers have used fMRI to assess neural activity and FC in the cortex of PD patients (Darcy et al., 2022; Chu et al., 2023). Structural MRI has also revealed that reduced thickness of the dorsolateral frontal and medial cortices is associated with the severity of gait freezing and that reduced gray matter volume in the presupplementary motor area and M1 is associated with gait variability (Mirelman et al., 2019). However, owing to the poor portability of MRI, its clinical translation and application are relatively difficult. Numerous studies using fNIRS have been conducted to explore the neurophysiological mechanisms of gait disturbances in PD patients and have shown good retest reliability (Ranchet et al., 2023; Chen et al., 2025), the results revealed that activity in the PFC is increased in PD patients when they are walking normally, indicating the mobilization of cognitive resources. The activity of the PFC becomes more pronounced with disease progression (freezing of gait) or when the difficulty of walking increases (such as by stepping over obstacles, turning, or changing posture). More specifically, in PD patients who have experienced gait freezing, the activity of the PFC is increased even in the absence of actual freezing episodes (Vitorio et al., 2020; Assad et al., 2022). The PFC plays an important role in walking and is involved in the formulation, organization, and response to the environmental processes of walking, which is a form of compensation (Holtzer et al., 2014). The aforementioned fNIRS results have demonstrated that as gait impairments in PD patients become more severe, the increase in gait variability is closely related to the dependence on the PFC. In addition to the PFC, gait disturbances in PD patients have been found to be associated with decreased SMA and M1 activity (Pelicioni et al., 2020; Leodori et al., 2024; Chen et al., 2025). M1 belongs to the direct postural regulation pathway and is activated when individuals perform less challenging walks, whereas the SMA and PFC belong to the indirect pathway and are activated when automatism is reduced or when cognitive compensatory resources are needed. The direct and indirect pathways work together to maintain gait stability (Beretta et al., 2020). Gait disturbances in PD patients are associated with decreased walking automaticity and require the mobilization of cognitive resources. Specifically, a shift from healthy automaticity to compensatory prefrontal executive control occurs (Mirelman et al., 2019). However, the regulatory mechanisms between these direct and indirect pathways and the subcortical direct and indirect pathways still need to be elucidated. Compared with studies using fMRI, studies using fNIRS measure cortical function while performing gait tasks, which can more intuitively reflect the neural activity of PD patients while they are walking (Mirelman et al., 2019; Beretta et al., 2020), revealing a decrease in the control of the cortical motor pathway over both the direct and indirect pathways, thus characterizing the functional impairment of the cortico-basal ganglia circuitry in patients with gait disorders and the underutilization of cognitive and motor resources. PD patients need to mobilize more regions of the brain to replenish lost cognitive and executive functions when performing complex tasks to better maintain a stable gait (Mirelman et al., 2019). By detecting activity in the PFC, M1 and SMA, fNIRS can be an ideal tool for mechanistic studies of gait disturbances in PD patients in the future (Leodori et al., 2024; Chen et al., 2025).

PD patients experience severe gait disturbances, typically characterized by slow starts, short stride lengths, rapid walking, and difficulty stopping (often referred to as panic gait). Traditionally, the diagnosis of gait disorders has relied primarily on the subjective judgment of neurologists during brief outpatient or inpatient consultations. In addition to pharmacological treatment, physical exercise is a major therapeutic strategy for managing gait disorders in PD patients. In recent years, advancements in sports medicine, such as motion assessment using wearable motion sensors combined with algorithmic models, have provided hope for the objective diagnosis of gait disorders. These developments also facilitate the customization of individualized neuromodulation therapies and exercise training prescriptions, which can be regularly adjusted based on recovery progress (Burtscher et al., 2024; van Midden et al., 2024). Despite the increasing availability of these sports medicine methods, investigations into the underlying neural mechanisms remain necessary. Studies have indicated that impairments in the cortico-basal pathway may be a potential mechanism underlying gait difficulties in PD patients (Beretta et al., 2020; Anjum et al., 2024; Bove et al., 2024; Nakamura, 2025). The cortico-basal pathway is a functional subregion known as the motor circuit, comprised of basal ganglia structures, the thalamus, and the motor and premotor cortices. This motor circuit includes both direct and indirect pathways. The direct pathway originates in the striatum and projects to the substantia nigra pars reticulata and the internal globus pallidus, while the indirect pathway originates in the striatum and projects to the external globus pallidus, which then projects to the subthalamic nucleus and subsequently to the substantia nigra pars reticulata and internal globus pallidus. These two pathways intersect and complement each other and are regulated by the motor and premotor cortices, which work together to maintain gait stability (Mirzac et al., 2023; Steiner et al., 2024). Previous studies have documented the characteristic neural activities of these direct and indirect pathways, which are considered markers of PD (Darcy et al., 2022; Lofredi et al., 2023). However, these studies have primarily focused on abnormalities in the basal ganglia, with insufficient attention given to impaired cortical function, and most have been preclinical studies conducted in nonhuman primates. Some researchers have performed fMRI to assess neural activity and functional connectivity in the cortex of PD patients (Darcy et al., 2022; Chu et al., 2023). Structural MRI has revealed that reduced thickness of the dorsolateral frontal and medial cortices is associated with the severity of gait freezing, and that reduced gray matter volume in the presupplementary motor area and M1 correlates with gait variability (Mirelman et al., 2019). However, due to the poor portability of MRI, its clinical translation and application are relatively challenging. Numerous studies using fNIRS have been conducted to explore the neurophysiological mechanisms underlying gait disturbances in PD patients, demonstrating good retest reliability (Ranchet et al., 2023; Chen et al., 2025). These results indicate that activity in the PFC increases in PD patients when they walk normally, suggesting the mobilization of cognitive resources. The PFC activity becomes more pronounced with disease progression (such as freezing of gait) or when walking difficulty increases (e.g., stepping over obstacles, turning, or changing posture). More specifically, in PD patients who experience gait freezing, PFC activity is heightened even in the absence of actual freezing episodes (Vitorio et al., 2020; Assad et al., 2022). The PFC plays a crucial role in walking, involved in the formulation, organization, and response to the environmental processes of walking, which serves as a compensatory mechanism (Holtzer et al., 2014). The aforementioned fNIRS findings demonstrate that as gait impairments in PD patients worsen, the increase in gait variability is closely related to the reliance on the PFC. In addition to the PFC, gait disturbances in PD patients have been associated with decreased activity in the SMA and M1 (Pelicioni et al., 2020; Leodori et al., 2024; Chen et al., 2025). M1 is part of the direct postural regulation pathway and is activated during less challenging walks, while the SMA and PFC belong to the indirect pathway and are activated when automaticity is reduced or when cognitive compensatory resources are needed. The direct and indirect pathways work in tandem to maintain gait stability (Beretta et al., 2020). Gait disturbances in PD patients are associated with decreased walking automaticity and necessitate the mobilization of cognitive resources. Specifically, there is a shift from healthy automaticity to compensatory prefrontal executive control (Mirelman et al., 2019). By detecting activity in the PFC, M1 and SMA, fNIRS can be an ideal tool for mechanistic studies of gait disturbances in PD patients in the future (Leodori et al., 2024; Chen et al., 2025).

From the above discussion, we can see that using fNIRS to measure cortical neural activity while performing gait tasks (such as simple walking or stepping over obstacles) can help explain the reasons for gait disorders in PD patients from the perspective of neural mechanisms. However, the current applications are still insufficient. Measuring cortical neural activity with fNIRS alone is not enough to fully explain the functional impairment of the cortico-basal circuitry. Furthermore, the retest reliability of fNIRS data needs to be established; this requires a large database that includes gait characteristics, cognitive levels, baseline physiological features, and previous treatment histories of each patient to jointly formulate standards for fNIRS data collection and analysis methods. In the future, fNIRS should be combined with EEG and incorporated into wearable motion sensors to measure the motor characteristics of gait disorders in PD patients and the corresponding changes in the cortico-basal circuitry. Notably, such scanning protocols must be standardized to facilitate cross-site data aggregation, thereby promoting reproducibility and generalizability. With the help of machine learning algorithms, the neural activity and gait variability characteristics of the brain should be analyzed to identify the key nodes of neural impairment in gait disorders. This will enable the selection of targeted treatment approaches and the formulation of individualized exercise training or other treatment plans.

#### New functional near-infrared spectroscopy metrics for exploring the mechanisms of gait disturbances in patients with Parkinson’s disease

In addition to recording alterations in brain activity in PD patients during walking tasks, other computational metrics derived from fNIRS can also be applied to PD research. In a follow-up study, a pattern of HbO_2_ variability in the PFC was observed during normal and dual-task walking in patients with PD. HbO_2_ variability was higher in the PFC during dual-task walking than during normal walking, and the increase in variability was significantly lower in PD patients compared to the healthy population. Notably, when multiple dual-task walks were performed, HbO_2_ variability in the PFC decreased in the healthy population but did not decrease in PD patients (Maidan et al., 2022). HbO_2_ variability is a response to changes in brain activity during different challenges. When faced with complex tasks, an increase in variability corresponds to a flexible adaptation to environmental demands. When trained repeatedly, a decrease in variability is related to brain function stability. Compared with the healthy population, PD patients exhibit decreased variability (including both flexibility and stability) (Maidan et al., 2022). Brain activity is inherently variable, and this variability provides deep insights into brain function. Compared with stable HbO_2_ levels, variable HbO_2_ levels reflect the brain’s ability to shift flexibly in response to different tasks and to maintain neural stability between tasks (Maidan et al., 2022). These findings offer new insights into the role of the PFC in the walking process of PD patients. The sources of such variability remain unclear. However, findings from other studies generally indicate that greater task variability optimizes flexible and adaptive responses under uncertain conditions (Zhao et al., 2015; Wang et al., 2024a, b). Additionally, the value of assessing variability lies in its ability to reflect the delicate balance between flexibility and stability in the nervous system: flexibility is demonstrated by comparing tasks of different difficulty levels (such as normal walking and dual-task walking in this study), while stability is demonstrated by comparing repeated tasks. The inability of PD patients to increase flexibility during dual-task processing or to enhance stability after repeated tasks may reflect the disease’s impact. Variability can also be assessed using fMRI (Nomi et al., 2017; Wang et al., 2024a, b), but the use of fNIRS allows for measurement during actual walking, providing a more intuitive reflection of the decline in PFC variability in PD patients. Lu et al. (2022) used fNIRS to measure HbO_2_ and HbR responses during dual-task walking in healthy controls and PD patients. They defined three brain states: dilated, contracted, and intermediate. The measurements were subsequently augmented with temporal parameters to quantify brain state transitions. The results revealed that the acceleration and angle of transitions were significantly lower in PD patients than in healthy controls (Lu et al., 2022). The human brain is a complex dynamic system that supports smooth and continuous transitions between states that underlie cognitive and motor functions. These brain state transitions respond to the brain’s ability to process information in time and space (Shine et al., 2019). The characteristics of brain state transitions are related to the degeneration of brain functions in PD patients (Lu et al., 2022).

In this section, we describe two main methods for measuring “dynamic” neural activity using fNIRS data. Compared to “static” brain activity and FC, this type of dynamic activity is more representative of the brain’s normal state, as the human brain never remains in an absolutely static state. Measuring variability and brain state transitions is essential for characterizing the brain’s response to environmental changes. However, currently, these two methods primarily focus on describing brain activity. Regarding dynamic FC, some researchers have already utilized fNIRS data in the context of autism. Nevertheless, fNIRS-measured dynamic FC data for PD patients are still lacking (Wan et al., 2024). In the future, additional dynamic brain activity and FC analysis methods may also be employed to explore neural activities related to gait disorders in PD patients.

#### Application of functional near-infrared spectroscopy in assessing movement disorders in patients with Parkinson’s disease

In addition to gait disturbances, patients with PD experience a variety of movement-related impairments, including resting tremors, muscle rigidity, bradykinesia, and balance issues. The emergence of these movement-related impairments indicates functional impairment in the neuromuscular pathways of PD patients, which are responsible for conveying motor commands between the muscles and the brain. This can lead to changes in the coupling between the brain and the body, also known as cortico-muscular coupling. Cortico-muscular coupling typically refers to the correlation between cortical activity and muscle contraction, as measured by electromyography (Abtahi et al., 2020; Houston et al., 2024). Therefore, physical activity signals must be synchronized with brain activity for researchers to better understand movement-related impairments. In previous studies, EEG or MEG has often been used to characterize cortical activity. However, due to the high sensitivity of these devices to movement artifacts, their use has been limited during measurements involving significant movement (Mujunen et al., 2022; Corsi, 2023). Combining fNIRS and EEG with physical activity signals (such as the MoCap system and WearUp glove) allows for more accurate differentiation between PD patients and healthy controls (Abtahi et al., 2020). Future advances in synchronizing brain and body activity should be facilitated by the use of more standardized measurement procedures and the development of standardized computational methods in larger populations. Additionally, extracting dynamic brain functional connectivity metrics during walking can improve the diagnosis of PD (Lu et al., 2023).

#### Application of functional near-infrared spectroscopy in the evaluation of new treatment methods for Parkinson’s disease

Patients with PD generally adopt treatments such as pharmacotherapy or rehabilitation training to control motor symptoms and maintain gait stability, enabling them to meet the demands of daily living. Based on the aforementioned effects of PFC function on gait in PD patients, enhancing the efficiency and potential of neural function should be a focus in PD treatment. Specifically, during daily walking, reduced PFC activation may be sufficient to meet basic demands. However, when facing complex environments or navigating obstacles, PFC activity should increase to meet higher cognitive demands (Stern, 2017). To achieve this, it is not enough to merely address the manifestations of motor impairments. Studies have confirmed that cognitive training combined with motor training is more effective at improving gait stability in PD patients than motor training alone (Mirelman et al., 2016; Johansson et al., 2023). These findings suggest that enhancing neural function in the PFC may benefit PD patients. One study revealed that PFC activity is reduced when tDCS is applied to the primary motor cortex (M1) in PD patients (Beretta et al., 2020). Compared with exercise alone, exercise combined with cognitive training further reduces PFC activity in PD patients while they are walking. This reduction in PFC activity is associated with fewer falls after training, indicating that during normal walking, the involvement of the indirect pathway is reduced, promoting a shift toward direct pathway-dominant automated walking in PD patients. In another study, applying tDCS to modulate PFC activity during aerobic exercise training in PD patients improved gait variability, processing speed, and walking execution control. Increased PFC activity in the stimulated hemisphere was also observed using fNIRS (Conceicao et al., 2021). These findings suggest that motor-cognitive training can help PD patients fully mobilize their PFC resources when coping with complex walking environments, thereby providing more effective compensation for the impaired walking automation mentioned earlier (Maidan et al., 2018). The aforementioned study used fNIRS as an assessment tool, providing direct evidence of the role of changes in PFC activity in improving gait impairments in PD patients. Previous studies have also utilized fMRI as an assessment tool (Fu et al., 2024; Qiu et al., 2024). However, despite the convenience and affordability of fNIRS, its widespread application has been significantly limited. Current research primarily uses fNIRS before and after treatment. If it could be used synchronously during stimulation, it would better highlight the advantages of fNIRS in real-time monitoring and feedback. In addition to the neuromodulation therapies mentioned, incorporating cholinergic-enhancing drugs such as donepezil with dopamine therapy for PD patients could significantly reduce PFC activity, promote gait recovery, and increase accuracy on concurrent cognitive tasks (Vitorio et al., 2021). In summary, current therapeutic approaches for PD should aim to improve basal ganglia function, remodel the cortico-basal pathway, reduce the implementation–attention resource burden, and increase the availability of cognitive resources, thereby enhancing gait and posture (Maidan et al., 2018; Beretta et al., 2020; Orcioli-Silva et al., 2020, 2021; Conceicao et al., 2021; **Additional Table 4**).

**Additional Table 4:** Application of fNIRS in exploring new therapeutic techniques for patients with Parkinson's disease

Study	Treatment methods	Research population	Stimulus area	Experimental design	Gait change	Primary endpoint events	Brain activity and functional connectivity results
Conceicao et al., 2021	tDCS	PD patients	PFC	A randomized, double-blinded, sham-controlled crossover study: different tDCS conditions (active- or sham-tDCS)	Active tDCS decreased step time variability, shortened simple and choice reaction times.	Spatiotemporal gait parameters, cognitive functions, and PFC activity while walking was assessed before and immediately after each session.	Active tDCS increased PFC activity.
Beretta et al., 2020	tDCS	PD patients	M1	A randomized, double-blinded, sham-controlled crossover study: different tDCS conditions (active- or sham- tDCS)	Active tDCS reduces time to regain equilibrium posture.	Lower limb electromyography and center of pressure parameters	Active tDCS decreased PFC activity.
Maidan et al., 2018	virtual reality	PD patients	None	A randomized control trial: treadmill training with virtual reality group and treadmill training group	Both groups improved gait efficiency and success over obstacles.	Brain activity of PFC	The treadmill training group increased PFC activation and the combined group decreased PFC activation.
Vitorio et al., 2021	Dopaminergic and cholinergic therapy	PD patients	None	A single-site, randomized, double-blind crossover trial: levodopa + donepezil or levodopa + placebo treatments	Spatiotempor al measures of turning improved with both treatments. The accuracy in the concurrent cognitive task improved only with levodopa + donepezil.	Brain activity of PFC	Levodopa + donepezil led to a large reduction in PFC activity.
Orcioli-Silva et al., 2020	Dopaminergic therapy	PD patients	None	A case-control study: PD group and healthy controls	Dopaminergic therapy increased in both step length and velocity.	Brain activity of PFC	Dopaminergic therapy increased PFC activation during dual task walking.
Orcioli-Silva et al., 2021	Dopaminergic therapy	PD patients	None	A case-control study: PD group and healthy controls	Dopaminergic medication increased step length and step velocity	Brain activity of PFC	Dopaminergic therapy increased PFC activation during obstacle avoidance walking.

This table presents fNIRS applications in PD therapeutics, highlighting its value in exploring novel interventions and evaluating treatment efficacy. fNIRS: Functional near-infrared spectroscopy; M1: primary motor cortex; PD: Parkinson's disease; PFC: prefrontal cortex; tDCS: transcranial direct current stimulation.

Unlike gait impairments, the tremor symptoms of PD patients typically do not improve with dopaminergic treatment. Anticholinergic medications may provide significant relief from tremor symptoms; however, their use is associated with an increased risk of cognitive decline and dementia in PD patients (Bloem et al., 2021). Because of this potential risk, many PD patients are indecisive when it comes to choosing anticholinergic medications. Therefore, objective and intuitive indicators are necessary to demonstrate the changes in brain activity caused by anticholinergic treatment. Wang et al. (2022a, b) utilized fNIRS to observe brain activation in the PFC of PD patients at rest and during the performance of a verbal fluency test (VFT). They found that brain activity was significantly lower in PD patients treated with anticholinergic medications compared to those who were not treated with these medications. These findings suggest that anticholinergic treatment may cause PFC dysfunction in PD patients, attenuating neural activity related to cognition and executive function. A review of the literature revealed that previous research on the impact of anticholinergic treatment on the brain has predominantly focused on dementia or elderly populations, primarily utilizing fMRI for measurements (Meng et al., 2022). We propose that using fNIRS to assess brain activity during anticholinergic treatment in PD patients represents a method with significant potential. Magnetic resonance imaging-guided focused ultrasound surgery (MRgFUS) is an emerging technology that has recently shown promise as a reliable treatment option for the tremor symptoms of PD patients (Weintraub et al., 2017). One study revealed that cortical hemodynamics measured by fNIRS improved after MRgFUS treatment in PD patients with tremor symptoms, resulting in improved brain activity, neural network remodeling, and symptom alleviation (Gurgone et al., 2024). To date, most studies on the mechanisms of MRgFUS treatment have used fMRI to assess brain function (Kociuba et al., 2023; Arcadi et al., 2024). However, relatively little data exist regarding the effects of MRgFUS on the cortical level of the motor network during the performance of motor tasks. In this context, the advantages of fNIRS become apparent, as it can be used while subjects perform complex tasks. Other benefits include reduced electrical noise, user-friendliness of the equipment, and portability, which enables convenient applications for pre-treatment and post-treatment monitoring in outpatient settings or even at the bedside. While fNIRS does have some disadvantages—such as the inability to detect deep brain functions and relatively poor spatial resolution—it still holds promise as a powerful tool for managing treatment and evaluating the tremor symptoms of PD patients during rehabilitation.

Some PD patients experience autonomic nerve disorders, which primarily manifest as lower urinary tract dysfunction, particularly overactive bladder (OAB) (Ogawa et al., 2017). Hou et al. (2023) applied fNIRS to PD patients with comorbid OAB and reported that the DLPFC is hyperactivated during the VFT, and that functional connectivity (FC) between the bilateral brain regions is increased in the resting state. Only a few studies have previously described the relationship between PFC dysfunction and OAB, and there is a lack of validation from functional neuroimaging (Walter et al., 2006; Hou et al., 2023). Such studies deepen our understanding of the nonmotor symptoms of PD and suggest potential alternative treatments. However, current studies with a cross-sectional design can only describe the association between PFC dysfunction and OAB. In the future, it will be necessary to combine molecular biological methods with longitudinal clinical trials to explore the role of PFC dysfunction in the pathogenesis of OAB.

PD is a complex neurodegenerative disease, and its pathophysiological mechanisms are still not fully understood. Previous studies have applied fNIRS to explore pathological damage loops associated with gait in PD patients (Wang et al., 2022a; Gurgone et al., 2024). From a therapeutic perspective, fNIRS may be performed to help explain the neural mechanisms of various treatments. In the future, fNIRS should be used in more studies to identify reliable biomarkers and new therapeutic targets for the nonmotor symptoms of PD, such as cognitive decline, mood disorders, olfactory decline, and autonomic disorders (**Additional Figure 2**).

### Application of functional near-infrared spectroscopy in dementia

#### Early detection and diagnosis of Alzheimer’s disease

AD is currently the most common type of dementia in clinical practice. Early diagnosis and treatment are paramount for improving the prognosis of AD patients (Rajan et al., 2021; Jack et al., 2024; Veitch et al., 2024). The International Working Group on AD has proposed significant and progressive situational memory deficits as the core criteria for AD (Lemos et al., 2015). Mild cognitive impairment (MCI) primarily leads to cognitive decline, and individuals with MCI are significantly more likely to develop AD than cognitively normal individuals. Early recognition and treatment of MCI can greatly enhance preventive effects against AD (Gauthier et al., 2006; Amrapala et al., 2023; Zhou et al., 2023). Traditionally, the diagnosis of AD and MCI relied on detailed clinical evaluations that included comprehensive cognitive tests (Ding et al., 2024). However, these assessments can be time-consuming and expensive, and medical records may increase the likelihood of classification errors. In recent years, advancements in artificial intelligence and machine learning have led many studies to utilize big data models to identify neuroimaging biomarkers for AD and MCI. For instance, PET has been pereformed to measure amyloid-β (Aβ) deposition, while magnetic resonance imaging (MRI) has been used to quantify brain atrophy. Among these methods, convolutional neural network algorithms based on deep learning have achieved the best performance, with a weighted average accuracy of 89% (Frizzell et al., 2022; Gao et al., 2023). However, these computational methods based on big data models have not yet been widely applied in population studies (Gauthier et al., 2006; Ding et al., 2024). Additionally, MRI and PET are expensive and typically available only in advanced medical institutions. Therefore, there is an urgent need for cost-effective and convenient technologies for large-scale screening of MCI and AD. Previous studies have started to utilize fNIRS to measure brain activity for screening MCI patients in the population (Kim et al., 2021; Ding et al., 2024). Results have indicated that the frontal, temporal, precentral, and parietal lobes are strongly associated with cognitive impairment (LiRui et al., 2020). Remote connections involving the PFC and OC may serve as reliable biomarkers for recognizing MCI (Zhang et al., 2022). Park (2023) reported that the activity of the left PFC demonstrated the highest sensitivity and specificity for identifying MCI, surpassing even the widely used Korean version of the Montreal Cognitive Assessment (AUC: 0.952 *vs*. 0.902). The application of multidimensional fNIRS has extended to MCI screening (Zhang et al., 2023). Kim et al. (2021) used this approach to identify neural loop markers for the PFC, and subsequent validation with fNIRS revealed that this refined network could accurately differentiate between healthy individuals and those with MCI or AD. The classification accuracy of fNIRS in distinguishing MCI patients from healthy individuals ranges from 70% to 95%, which is comparable to the results of big data models based on PET and MRI (Kim et al., 2021; Park, 2023). Previous studies have also explored the role of fNIRS in early AD recognition (Ung et al., 2020; Yang et al., 2020). A good biomarker for characterizing AD should be evaluated for sensitivity to specific cognitive impairment screening methods. Given the degenerative nature of AD, biomarkers must be validated across a wide range of pathomechanisms for ongoing disease monitoring. Therefore, using brain networks with uncorrelated variance removal—i.e., refined networks—can enhance classification performance for AD. The application of fNIRS combined with EEG to detect brain activity and neurovascular coupling during working memory tasks and free and cued selective reminder tasks has significantly aided in identifying patients with early AD (Perpetuini et al., 2018). Additionally, fNIRS has revealed normal neural compensation in the PFC of the MCI population and decreased neural compensation in the AD population (Ung et al., 2020). These results suggest the great potential of fNIRS for detecting MCI and early AD patients. Improving methods for analyzing the temporal feature map of fNIRS signals through machine learning could further facilitate early screening for cognitive impairment (Yang et al., 2020). Furthermore, compared to PET and MRI, fNIRS can measure neural activity during cognitive tests, which likely contributes to its increasing popularity in recent years.

Olfactory dysfunction is an early symptom of many neurodegenerative diseases, including AD and MCI, and may precede cognitive decline. Its pathological mechanism may be related to the deposition of α-synuclein, hyperphosphorylated tau protein, and neurofilaments in the olfactory epithelium, olfactory bulb/tract, primary olfactory cortex, and secondary targets (Attems et al., 2014; Pacyna et al., 2023). Recent research on olfactory dysfunction has focused on its potential as an early biomarker for diagnosing neurodegenerative diseases and tracking disease progression. This potential has been confirmed by studies using the Sniffin’Sticks identification test and neuroimaging techniques such as MRI, EEG, and PET (Dong et al., 2023; Liao et al., 2024b; Chang et al., 2025). In recent years, olfactory-stimulated fNIRS has been developed to explore olfactory function. Kim et al. reported that differences in olfactory-stimulated activity in the orbitofrontal cortex, as detected by fNIRS, are significantly associated with cognitive impairment, and this association becomes even more pronounced as cognitive impairment worsens. Compared with amyloid PET and fMRI, fNIRS has shown greater sensitivity for the diagnosis of MCI and AD (Kim et al., 2022). Subsequent calculations using machine learning methods yielded significantly higher accuracy than traditional statistical methods, with fNIRS providing the most reliable data (Kim et al., 2023). In addition to olfactory function, deficits in visuospatial processing in the parietal cortex are a reliable risk factor for AD (Ilardi et al., 2022). Haberstumpf et al. (2022) reported significantly lower activation of the parietal cortex in MCI patients compared with healthy individuals while performing a clock-hand-angle discrimination task. These findings corroborate previous hypotheses that hemodynamic alterations in visuospatial processing deficits are localized in the parietal cortex and may serve as early markers of AD. Studies on olfactory dysfunction and visuospatial processing deficits have shown that fNIRS can be combined with targeted task tests, such as the olfactory-stimulated and clock-hand-angle discrimination tasks used here, to reveal the neural mechanisms underlying these functional impairments prior to cognitive decline (Ilardi et al., 2022; Kim et al., 2022). fNIRS technology offers a potential noninvasive and less expensive alternative to fMRI, PET, and questionnaires for diagnostic purposes or treatment monitoring in real-world clinical settings. However, research on fNIRS is limited by relatively small study populations, low-evidence study designs, and a lack of standardization regarding the types of stimuli and methods of interpretation.

#### Determination of dementia type

Many types of dementia, including AD, frontotemporal dementia (FTD), and vascular dementia, have been identified, each with different etiologies and pathogenesis. However, the overlap of cognitive symptoms in the early stages complicates the identification of the specific type of dementia (Hodges et al., 2004; Guo et al., 2024; Reuben et al., 2024). This limitation underscores the need for research on objective and reliable biomarkers to elucidate cortical mechanisms and assist in distinguishing between dementia types. The structural MRI manifestations of FTD and AD are similar, primarily characterized by significant atrophy of the frontal and temporal lobes (Whitwell et al., 2009; Risacher et al., 2023). fMRI can help researchers understand brain FC, brain activity, and acquired brain networks. According to fMRI data, the default network is severely impaired in AD patients but is less affected in FTD patients (Allen et al., 2007; Raji et al., 2022). Most fMRI measurements are recorded in the resting state, which complicates the integration of tasks into studies of brain function due to the application settings of fMRI (Virani et al., 2013; Chouliaras et al., 2023). In contrast, fNIRS provides task-state measurements that are more aligned with real-world neuropsychological contexts. While performing a VFT, healthy older adults showed increased activity in the frontoparietal cortex. AD patients exhibited similar results but to a lesser extent, whereas FTD patients showed activation in the frontopolar and Broca regions (Metzger et al., 2016). Older adults maintain cognitive function during brain aging through functional reorganization, which involves recruiting additional regions (e.g., frontotemporal and parietal lobes) for activity during cognitive tasks (Cabeza, 2002). This phenomenon is also observed in AD patients; however, FTD patients display a significantly different pattern of brain recruitment. This may indicate that frontal cortex dysfunction is more severe in FTD patients compared to those with AD. Specifically, the activation of Broca’s area in FTD patients suggests a loss of connection with the frontoparietal control network, leading to poor performance on the VFT (Nishida et al., 2013). From these studies, it is evident that fNIRS has the capability to distinguish between different types of dementia and can intuitively illustrate the differences in brain neural activity among them (Nishida et al., 2013; Metzger et al., 2016). However, relying solely on fNIRS to characterize the differences in brain function between patients with AD and those with FTD is somewhat limited. Future research should also consider whether genetic variations can affect brain function in patients with FTD.

Currently, fNIRS is used mainly for the early identification and diagnosis of dementia. For example, reliable AD biomarkers and methods for differentiating dementia types have been discovered. With the development of BCI and neuromodulation technologies, the treatment of dementia is also expected to be revolutionized by these emerging technologies. Moreover, new therapeutic targets can be discovered with the assistance of these technologies. Since fNIRS has been proven to have many promising associations with these technologies in patients with stroke and PD, the application of fNIRS in patients with dementia is also expected to be a breakthrough in this regard.

### Application of functional near-infrared spectroscopy in amyotrophic lateral sclerosis

ALS is a progressive motor neurodegenerative disease. The diagnosis of ALS is challenging due to the significant heterogeneity of its clinical manifestations and the overlap with many other neurological diseases. Early diagnosis during the course of the disease can improve patient prognosis, as timely intervention can slow disease progression (Goutman et al., 2022; Ilieva et al., 2023; McMackin et al., 2023). To facilitate early diagnosis, new ALS standards and scoring systems are necessary. Machine learning algorithms are assisting in the search for emerging diagnostic and prognostic biofluid biomarkers, imaging modalities, and electrophysiological measurements (such as neuroimaging and genetic engineering) (The Lancet Neurology, 2024). A comprehensive fMRI survey of the brain network revealed that as ALS progresses, FC within the brain network becomes increasingly tight. This change involves the entire brain rather than being focal (Sorrentino et al., 2018; Du et al., 2024). Moreover, such changes in brain networks may have a causal relationship with the onset and progression of ALS (Thompson et al., 2024). In addition to fMRI, other MRI sequences, as well as EEG and PET, may also serve as tools for the early diagnosis of ALS. In fact, there is currently an emphasis on integrating multimodal tools (Goutman et al., 2022). Using fNIRS, Borgheai et al. (2020) identified frontal functional network asymmetry as a potential neurotopological hallmark of ALS. Unlike other neuroimaging techniques that typically scan in a resting state, a characteristic of fNIRS is its ability to detect disruptions in the topology of the frontal functional brain network within activity-based paradigms. This supports the use of fNIRS as a bedside assessment method for determining the functional state of brain networks in patients with ALS.

As the disease progresses, ALS patients gradually lose all voluntary motor control. Assistive communication devices can improve quality of life and facilitate care by providing a certain degree of independence (Mercadante et al., 2023; Willett et al., 2023). BCIs are emerging technologies that encode the brain’s response to mental activity into signals that can be communicated. Motor imagery (MI) has been adopted as a BCI paradigm after ALS patients progress to the point where they are unable to perform true motor executive activities (Pfurtscheller et al., 1997; Card et al., 2024). Noninvasive BCI systems based on EEG have been the most widely used in the past, with the P300 visual event-related potential being the most commonly applied communication paradigm. However, as the ocular muscles of ALS patients become paralyzed, P300 vision-based BCIs are no longer effective (Pires et al., 2022). In this situation, fNIRS is considered to have great application prospects in the treatment of advanced ALS patients because it can be performed at the bedside and does not require patients to cooperate in completing specific tasks; it only requires MI. Borgheai et al. (2020) reported that an fNIRS-based BCI system combined with a single-trial visuospatial task allows patients with advanced ALS to communicate. These results show that the performance of ALS patients using an fNIRS-based BCI can be comparable to that of healthy cohorts using existing BCIs. Hosni et al. (2020) used fNIRS to quantify the specific spatiotemporal characteristics of hemodynamics in ALS patients who underwent MI. The results showed that all subjects could control the BCI via their hemodynamic responses during the MI task. Increased bilateral activity in the PFC, as well as in the frontal and primary motor cortices (PMC), was observed in almost all subjects, indicating brain region activity during ME, rather than actual muscle activity. These results are very encouraging, as they demonstrate the feasibility of fNIRS-based BCI systems in restoring communication and control for patients with severe motor impairments.

In recent years, nonmotor systems (behavioral and cognitive) have been shown to be affected in individuals with ALS. Executive dysfunction in these patients is a poor prognostic indicator. Patients who do not have cognitive impairment at the time of diagnosis may develop it later, and some cases of cognitive impairment are associated with worsening motor function (Goutman et al., 2022). The mechanisms underlying nonmotor deficits remain unclear, and the search for reliable biomarkers could complement objective indicators in patients with ALS, thereby improving current diagnostic and therapeutic techniques (Consonni et al., 2016). These nonmotor symptoms are often accompanied by structural and functional impairments in nonmotor areas of the brain. In recent years, advanced brain MRI and PET, along with emerging advanced spinal cord imaging applications, have facilitated significant progress. However, how to integrate imaging into clinical care at the individual patient level still requires further study (Vucic et al., 2018). With the advantage of being able to conduct measurements in natural settings, fNIRS has also attracted attention for exploring the neural mechanisms of nonmotor symptoms in individuals with ALS. Borgheai et al. (2019) used EEG-fNIRS to detect brain electrical activity and hemodynamic responses in ALS patients and healthy controls during the performance of a visuospatial task. The results revealed a significant reduction in both EEG and fNIRS activity in ALS patients compared with healthy controls. Additionally, a significant correlation between EEG signals and HbO_2_ levels was observed in healthy controls but not in ALS patients. Kopitzki et al. (2016) assessed damage to nonmotor areas in ALS patients using fNIRS and diffusion tensor imaging. Their findings indicated that homonymous FC was altered in ALS patients compared with healthy controls, and that FC in the anterior temporal cortex (ALT) was significantly associated with motor decline. Furthermore, the FC of the ALT was significantly correlated with the fractional anisotropy of the central corpus callosum and corticospinal tract, as measured by diffusion tensor imaging (Kopitzki et al., 2016). These results suggest the presence of neural damage in nonmotor regions in ALS patients, and that FC in the ALT may serve as a favorable biomarker. However, the aforementioned studies are primarily exploratory, focusing on characterizing phenomena rather than applications (Kopitzki et al., 2016; Borgheai et al., 2019). In the future, in conjunction with the previously discussed treatment of advanced ALS patients using fNIRS-BCI systems, these fNIRS-discovered biomarkers could serve as potential integrated control signals for BCIs, allowing ALS patients to communicate with the outside world. We summarize the key points regarding the application of fNIRS in AD and ALS in **Additional Figure 3**.

## Application of Functional Near-Infrared Spectroscopy in Epilepsy

### Application of functional near-infrared spectroscopy in monitoring epilepsy

For patients with status epilepticus, continuous scalp EEG monitoring is routinely used in the neurocritical care unit. However, preclinical studies have shown increases in cerebral blood flow, oxygen pressure, and metabolism in the epileptic brain (Bitar et al., 2024; Gettings et al., 2025). These findings indicate that epilepsy is not only a disease characterized by abnormal electrical activity but is also accompanied by vascular abnormalities. Therefore, incorporating neuroimaging techniques into EEG monitoring can aid in the diagnosis of status epilepticus and enhance the understanding of its pathophysiological mechanisms (Sinka et al., 2023; Fernandez-Torre et al., 2024). MRI and 18-fluorodeoxyglucose positron emission tomography (^18^F-FDG PET) have revealed increased cerebral blood flow and hypermetabolism during epileptic seizures (Bonduelle et al., 2023; Hobson et al., 2024). However, none of these modalities can be used for continuous monitoring at the bedside. fNIRS is currently the only noninvasive technique that allows for prolonged monitoring of brain hemodynamics. Previous studies have shown that seizures are associated with an increase in HbO_2_ levels and a decrease in HbR levels, with the duration of these changes positively correlated with seizure duration (Chavko et al., 2006; Zhao et al., 2007). Consistent with the results of other neuroimaging studies, cerebral blood flow and metabolism increase during status epilepticus (Kassab et al., 2021; Sasaoka et al., 2022). These findings demonstrate that prolonged monitoring of hemodynamic changes in patients with severe epilepsy is feasible and represents a new approach for understanding and managing abnormal EEG patterns. Additionally, the hemodynamic responses measured by fNIRS are greatest in the regions involved. This is significant for clinical practice, indicating that fNIRS not only aids in the diagnosis of status epilepticus but also enhances the ability to localize epileptic foci.

fNIRS has emerged as a valuable tool for monitoring hemodynamic changes in patients with epilepsy, particularly in instances where traditional EEG monitoring faces challenges. Unlike EEG, which primarily detects electrical activity, fNIRS provides insight into changes in blood flow and oxygenation levels in the brain, offering a complementary perspective on epileptic activity. Research indicates that fNIRS can detect alterations in the hemodynamic response—specifically, changes in HbO_2_ and HbR—prior to the peak activation observed on EEG. This capability allows for early localization of epileptic foci, which is crucial for effective treatment planning and intervention. In patients with temporal lobe epilepsy, fNIRS studies have reported significant hemodynamic changes, such as increased cerebral blood volume and HbO_2_ levels, alongside a biphasic response in HbR levels (initial decrease followed by an increase). These changes not only occur in the temporal cortex but also extend to contralateral regions, including frontal and parietal cortices, highlighting the interconnected nature of brain regions during seizures. Notably, these hemodynamic changes can persist beyond the duration of the seizure itself, suggesting that fNIRS can provide valuable information about the brain’s response to seizures and potential recovery processes. Overall, fNIRS is a promising approach for enhancing our understanding of epilepsy, enabling clinicians to monitor brain activity and hemodynamics in real time and potentially improving patient outcomes through more informed clinical decisions.

### Application of functional near-infrared spectroscopy in predicting epilepsy

Epilepsy prediction relies on monitoring systems that collect and analyze brain activity signals from patients with epilepsy. Seizures can be predicted when signals indicating a pre-epileptic state are detected. Most predictions are based on the collection of EEG signals (Ramgopal et al., 2014). A previous study using fMRI-EEG has shown that alterations in brain oxygenation during seizures occur within the first few seconds of the electrical seizure, suggesting that the BOLD signal changes recorded by fMRI precede the electrical signals detected by EEG (Hawco et al., 2007). However, synchronous EEG-fMRI still faces numerous challenges. fMRI equipment is relatively expensive and is typically available only in advanced medical institutions or research facilities. Additionally, fMRI requires trained personnel for data acquisition. During the fMRI acquisition process, patients must remain still, and the acquisition time is relatively long, usually around 1 hour.

The probability of fMRI detecting hemodynamic signals that precede electrical activity ranges between 23% and 50%, making long-term fMRI scanning impractical. As discussed in Section “Application of functional near-infrared spectroscopy in Parkinson’s disease,” fNIRS assesses changes in cortical hemodynamics, which can help detect status epilepticus and evaluate its effects on brain oxygenation (Nguyen et al., 2013). Consequently, many researchers have begun exploring the potential of fNIRS for seizure prediction. Pellegrino et al. (2016) demonstrated that fNIRS could detect a sustained and strong hemodynamic response for 30 seconds prior to interictal epileptic discharges—spontaneous and transient epileptic events that precede seizures. These findings suggest that, compared to EEG, fNIRS can detect pre-status epilepticus signals earlier and holds greater promise for predicting epilepsy. Rosas-Romero et al. (2019) constructed a convolutional neural network analysis framework to evaluate the reliability of fNIRS in predicting epilepsy. Their results indicated that convolutional neural network-based analysis of fNIRS signals achieved a performance index of over 95% for epilepsy prediction. Notably, combining HbO_2_ and HbR levels yielded better predictive accuracy than using either parameter alone.

Based on current studies, fNIRS demonstrates significant potential for monitoring brain hemodynamics in patients experiencing status epilepticus (Pellegrino et al., 2016; Rosas-Romero et al., 2019). Notably, fNIRS has the advantage of detecting pre-status epilepticus signals earlier than traditional EEG. However, it is important to note that existing studies in this area have primarily been exploratory, and the findings have not yet been validated in actual clinical settings (Pellegrino et al., 2016; Rosas-Romero et al., 2019). To advance this field, there is a pressing need for more standardized clinical trials to validate these findings and establish reliable biomarkers for epilepsy prediction. Looking ahead, integrating fNIRS with EEG for multimodal data fusion presents a promising avenue for enhancing the accuracy of brain signal decoding in complex tasks (Chen et al., 2024c). This approach capitalizes on the complementary strengths of both techniques, which could lead to improved performance in BCI systems, particularly for motor imagery decoding. Furthermore, such integration may also facilitate advancements in neuromodulation therapies. By providing real-time feedback on both electrical activity and hemodynamic signals, this combined approach can support self-regulation of brain activity in epilepsy, promote cognitive rehabilitation, and potentially contribute to the development of smart device control systems. In summary, the application of fNIRS in the context of epilepsy holds great promise, but future research must focus on rigorous clinical validation and the integration of multimodal data to unlock its full potential. Key points regarding the application of fNIRS in epilepsy are illustrated in **Additional Figure 4**.

## Application of Functional Near-Infrared Spectroscopy in Traumatic Neurological Disorders

### Application of functional near-infrared spectroscopy in spinal cord injury

Every year, hundreds of thousands of people worldwide suffer a spinal cord injury (SCI), with 90% of cases caused by trauma. SCI leads to the disruption of connections between the brain and the spinal cord, resulting in devastating and permanent nerve damage, including sensory and motor impairments, abnormal reflexes, and autonomic dysregulation (Fang et al., 2023; Qin et al., 2023). Although the life expectancy of patients with SCI has dramatically increased over the past few decades, a significant proportion of individuals are left with long-term paralysis and lifelong disabilities (Ma et al., 2024; You et al., 2024).

In recent years, the development of new methods in molecular biology, materials science, genetic engineering, and computer science has led to promising results in neuromodulation technologies, which aim to improve the prognosis of patients with SCI. Some phase I and II clinical trials have already begun (Tian et al., 2023; Zhou et al., 2024a, b). The structure and function of the motor cortex in the brain and motor circuits below the level of a SCI are preserved in patients with SCI. Neuromodulation technologies applied to SCI can decode signals from the motor cortex to activate motor circuits below the injury level, thereby promoting the recovery of motor function and voluntary movement. Preclinical and basic research has demonstrated that BCI technologies can facilitate the recovery of motor function in patients with SCI (Samejima et al., 2021; Willett et al., 2021). As discussed in the context of stroke patients, one of the main obstacles to applying BCIs in SCI patients is characterizing motor region reorganization. EEG is the most commonly used method for decoding motor signals in BCIs. Several trials have demonstrated the possibility of controlling limb movements in SCI patients using EEG-based BCI systems through MI (Xu et al., 2022; Mirzabagherian et al., 2023). Despite its limitations, fNIRS is less susceptible to electrical noise from surrounding equipment, muscle contractions, or head movements compared to EEG. Its portability and low cost highlight the potential for fNIRS to contribute to the study of motor cortex control. Koenraadt et al. (2014) used fNIRS to explore brain activity in the motor cortex during foot movements in patients with SCI. They reported significant HbO_2_ and HbR responses in the contralateral primary motor cortex (M1) when SCI patients attempted foot movements compared with hand movements, indicating that fNIRS can measure neural activity in the motor cortex regions that were previously responsible for controlling limb movements below the level of injury. These results confirm that fNIRS can play a role in characterizing motor cortex activity in SCI patients, potentially supporting subsequent BCI studies to promote the application of fNIRS in SCI (Koenraadt et al., 2014; Deepti Karunakaran et al., 2023). More studies should be conducted to standardize fNIRS scanning parameters and BCI performance parameters (Chen et al., 2024a, b, c; Dai et al., 2024).

In addition to motor impairment, SCI patients are at a much higher risk of autonomic dysfunction, which is closely related to a decline in cerebrovascular reactivity (CVR). CVR refers to the ability of blood vessels to dilate or constrict in response to vasoactive stimuli and represents the vascular health of the brain (Pinto et al., 2020; Sleight et al., 2021). At present, relatively few studies have investigated the changes in CVR after SCI (Chen et al., 2024a, b; Kilgore et al., 2024). Weber et al. (2024) reported that, compared with healthy controls, SCI patients have a prolonged SVR delay time. However, the study by Weber et al. (2024) had a small sample size, which may be attributed to the challenges SCI patients face in completing demanding MRI scans. Recently, Chen et al. (2024a, b, c) used fNIRS to measure the differences in CVR between patients with SCI and healthy controls matched for sex and age. The results revealed that CVR in the right inferior parietal lobe of SCI patients was significantly delayed, with the delay time negatively correlated with the duration since injury. Further subgroup analysis revealed that the delay in CVR in patients with SCI was only associated with the HbR index; this can be considered a unique finding of fNIRS, as fMRI cannot distinguish the dynamic changes in HbR from the dynamic changes in HbO and HbT measured for CVR (Biondetti et al., 2023).

### Application of functional near-infrared spectroscopy in traumatic brain injury

Traumatic brain injury (TBI) is a common cause of death and disability, resulting in serious social and economic burdens worldwide (Dams-O’Connor et al., 2023; Lavinio et al., 2024; Cook et al., 2025). Traumatic cerebral vascular injury (TCVI), which often occurs after TBI, plays an important role in the pathological mechanism leading to functional deficits and chronic disability and may become a potential therapeutic target (van Hameren et al., 2023; Hao et al., 2024; Singh et al., 2024; Whitehead et al., 2024).

In the past, neuroimaging studies of TCVI following TBI were relatively rare and primarily focused on contact sport athletes or patients in inpatient trauma wards (Zhao et al., 2023; Whitehead et al., 2024). The studies by scholars had small sample sizes and focused on more severe cases (Rauchman et al., 2023; Fouche et al., 2024). Therefore, the findings are not generalizable to the broader population of chronic TBI patients. Low et al. (2024) used MRI to assess the occurrence of TCVI in chronic TBI patients, and indeed, the potential detection rate increased. However, that study was conducted using a population of white individuals with higher levels of education, which, in addition to the high cost of MRI, limits the generalizability of the results to a broader population. Amyot et al. (2020) detected a substantial decrease in CVR in patients with chronic TBI compared with healthy controls using both fMRI and fNIRS techniques. The global CVR acquired by fNIRS was consistent with the fMRI BOLD signal, reflecting the presence of severe TCVI in TBI patients. Compared with the expensive and inconvenient characteristics of fMRI, fNIRS data acquisition is inexpensive and easy to repeat and has great potential in the development of TCVI biomarkers (Amyot et al., 2020).

Severe TBI can result in disabling disorders of consciousness. However, related evidence-based treatments are lacking (Laureys et al., 2010; Snider et al., 2023; Johnson-Black et al., 2025). Straudi et al. (2023) performed 10 tDCS anodic stimulations of bilateral PMC areas in patients with disorders of consciousness and examined hemodynamic changes in the brain after each stimulation. The results revealed increased brain activity after a single tDCS and the suppression of the inflammatory response after multiple tDCSs, contributing to functional recovery. These findings provide new biological targets for fNIRS-assisted tDCS in the rehabilitation of severe TBI patients with disorders of consciousness. Key findings regarding the use of fNIRS in TBI and SCI patients are shown in **Additional Figure 5**.

## Application of Functional Near-Infrared Spectroscopy in Other Neurological Disorders

In addition to the abovementioned diseases, fNIRS has been applied in other neurological disorders, and the relevant key findings are shown in **Additional Figure 6**.

### Application of functional near-infrared spectroscopy in migraine

Migraine is a common disabling condition that affects 15.2% of the population worldwide (Stovner et al., 2022). The mainstream treatment methods remain pharmacological, primarily involving serotonin and calcitonin gene-related peptide (CGRP) antagonists (Labastida-Ramirez et al., 2023; Messina et al., 2023; Mitsikostas et al., 2023). Clinical trials have demonstrated the efficacy of monoclonal antibodies targeting CGRP in the treatment of migraine (Edvinsson et al., 2018; Li et al., 2023a, b; Puledda et al., 2023). However, the exact mechanism of action of anti-CGRP antibodies remains unknown. Evidence of altered excitability and FC in the visual cortex during migraine attacks has been reported, as confirmed by EEG and MRI studies (Lau et al., 2020; Christensen et al., 2022; Coppola et al., 2022; Karsan et al., 2023). To investigate whether CGRP can regulate the activity of the visual cortex, de Tommaso et al. (2022) combined EEG and fNIRS techniques to assess the efficacy and mechanism of galcanezumab-GCA (a monoclonal antibody against CGRP) in treating migraine patients. The results revealed that levels of HbO_2_ in the occipital cortex were elevated during visual stimulation in migraine patients before treatment but returned to normal after GCA treatment. These findings suggest that GCA exerts a therapeutic effect against migraine by ameliorating hemodynamic disturbances, thereby suppressing excessive visual cortical reactivity (Huang et al., 2012). Collectively, these results indicate that CGRP elicits a central response through peripheral regulation, which could aid in the development and application of migraine medications.

In addition to the visual cortex, dysfunction in the frontal cortex also plays a role in the pathogenesis of migraine. Zengin et al. (2025) used fNIRS to assess hemodynamic changes in the frontal cortex of migraine patients during cognitive tasks. Compared with healthy controls, migraine patients exhibited decreased PFC activity, an effect that was related to headache severity, attack frequency, and the number of migraine days per month. This finding suggests that migraine may be a chronic neurovascular uncoupling disorder, and the decrease in prefrontal activity may result from functional impairment.

### Application of functional near-infrared spectroscopy in dystonia

Dystonia is a movement disorder that is characterized primarily by involuntary muscle contractions that result in abnormal executive or twisting movements (Albanese et al., 2013, 2025; Shaikh et al., 2025). With the development of neuroimaging techniques, numerous studies have identified specific brain regions whose abnormalities lead to dystonia, often accompanied by the disruption of brain networks (Herold et al., 2018; Li et al., 2023a, b). Moreover, the extent of these brain network abnormalities frequently exceeds the regions associated with motor symptoms. Therefore, multimodal imaging studies are warranted to delineate underlying symptom-specific mechanisms and tailor individualized treatment approaches (Joutsa et al., 2023; Ellis et al., 2024). fMRI results have shown that dystonia may be associated with the increased activation of M1 or the sensory cortex (S1) (Jinnah et al., 2017; Li et al., 2023a, b). In a study by de Faria et al. (2020), fMRI results revealed increased cerebellar and occipital cortex activity, and fNIRS results revealed decreased frontal and somatosensory cortex activity in patients with right upper extremity dystonia performing a tapping task with the affected hand. In contrast, when a two-handed tapping task was performed, both fMRI and fNIRS revealed decreased frontal cortex activity. The results of that study show that fNIRS can complement fMRI and has potential in the study of dystonia (de Faria et al., 2020). Unlike fMRI, which collects data in a resting state, fNIRS allows brain function measurements to be recorded while motor tasks are being performed (Herold et al., 2018). Therefore, fNIRS has unique advantages in the exploration of dystonia and other movement disorders. Proa et al. (2021) used fNIRS to measure brain activity during a writing task in a population with right upper limb dystonia. The healthy group showed only contralateral M1 and S1 activation, and the dystonia group showed bilateral M1 and S1 region activation. The degree of activation was greater in the dystonia group than in the healthy group (Proa et al., 2021). Results from another study have revealed an increase in somatosensory processing and task-irrelevant motor muscle recruitment in dystonia patients (Braun et al., 2003). In the future, fNIRS signals could be used for real-time neurofeedback to modulate the activity of the sensorimotor cortex in dystonia patients, thereby promoting functional recovery.

### Application of functional near-infrared spectroscopy in dural arteriovenous fistulas

Dural arteriovenous fistulas (DAVFs) is an abnormal intracranial arteriovenous shunt disorder with major disabling complications, including intracerebral hemorrhage and nonhemorrhagic neurological deficits (NHNDs) (Tanaka, 2019). Intracerebral hemorrhage can quickly be recognized by typical clinical symptoms and computed tomography scans of the brain, and NHNDs include a group of neurological symptoms caused by venous blood congestion, such as cognitive decline, seizures, and dysphagia. In many cases, mild NHNDs are difficult to recognize (Racine et al., 2008). Senthilvelan et al. (2022) applied fNIRS to DAVF patients and reported decreased HbO_2_ levels in the PFC of the DAVF group compared with the healthy control group; after surgical embolization, HbO_2_ levels increased significantly and were correlated with improved Mini-Mental Status Examination scores. These results indicate that fNIRS enables the detection of brain activity in DAVF patients at the bedside and can be used to monitor treatment responses.

### Application of functional near-infrared spectroscopy in duchenne muscular dystrophy

Duchenne muscular dystrophy (DMD) is an X-linked disorder characterized by progressive muscle weakness and atrophy (Roberts et al., 2023; Mercuri et al., 2024; Vaillend et al., 2025). In patients with DMD, mutations in the genes encoding myotonic dystrophy proteins lead to reduced myofilm stability and the susceptibility of muscle fibers to contractile injury. Moreover, mutations in these genes also lead to the impaired signaling of dystrophy-related proteins, such as reduced neuronal-type nitric oxide synthase recruitment and reduced nitric oxide production, contributing to functional muscle ischemia during exercise (Duan, 2024; Hart et al., 2024; Rind, 2024). Currently, the 6-minute walk test (6-MWT) and the North Star Ambulatory Assessment are indicators for observing clinical symptoms and monitoring treatment, but both tests are highly subjective (McDonald et al., 2010). Weng et al. (2018) used fNIRS to determine the dynamic changes in muscle hemodynamics (gastrocnemius and forearm muscles) in DMD patients and healthy controls during the 6-MWT and venous occlusion test. The results revealed that during the 6-MWT, muscle oxygenation was impaired in DMD patients compared with healthy controls, and the venous occlusion test results suggested that muscle hypoplasia was associated with inadequate oxygenation. fNIRS can serve as a sensitive and objective technique for determining muscle microcirculation in DMD patients (Weng et al., 2018). The results of that trial suggest that fNIRS has great potential for clinical applications such as disease assessments and monitoring treatment efficacy in DMD patients. Previous studies also used contrast-enhanced ultrasound and perfusion MRI to assess muscle microcirculation (Kerwin et al., 2013; Xie et al., 2022, 2024). Compared with these techniques, fNIRS offers advantages for functional exercise studies, as it can be used to assess not only muscle perfusion but also muscle oxygenation (Weng et al., 2018). In other words, fNIRS provides technical support for patient-oriented measurements.

### Application of functional near-infrared spectroscopy in multiple sclerosis

Multiple sclerosis (MS) is the most common central nervous system immune disease in young people, with the main symptoms being gait impairment and cognitive dysfunction (Bagnato et al., 2024; Tafti et al., 2025). Previous studies have shown that patients with MS often experience a decline in the ability to perform cognitive and motor tasks (such as walking) (De Meo et al., 2021; Golabi et al., 2024). This decline may be related to the impaired function of the PFC. Compared with healthy controls, MS patients exhibit increased activation in the PFC during walking. This phenomenon may indicate that, owing to impaired PFC function in MS patients, the brain needs to recruit more regions to compensate (Hernandez et al., 2016; Zuppichini et al., 2023; Santinelli et al., 2024). Therefore, some scholars have used fMRI and structural MRI combined with psychological assessment to characterize changes in brain structure and neural activity in MS patients (Filippi et al., 2012; Al-Iedani et al., 2022; Hernandez et al., 2025). However, those studies mostly measured data from MS patients in a resting state, which cannot truly reflect the brain neural activity of MS patients while they are walking (Al-Iedani et al., 2022; Hernandez et al., 2025). To verify the changes in PFC neural activity during walking in MS patients, some researchers have used fNIRS to measure neural activity in MS patients and healthy controls while performing dual-task forward walking and single-task backward walking. Increased neural activity in all subregions of the PFC was observed during dual-task forward walking compared with single-task backward walking in both MS patients and healthy controls. However, that study did not observe statistically significant differences between MS patients and healthy controls. The 6-MWT is also a commonly used paradigm to assess walking in MS patients, and changes in gait parameters can be observed. However, fNIRS has not yet been used to detect changes in PFC activity in MS patients during the 6-MWT. This may be because the test does not induce functional compensation of the PFC (Broscheid et al., 2022), leading us to question the ability of fNIRS to characterize neural activity in MS patients. However, a turning point has occurred in the past 2 years, with some researchers using ML methods to assess neural activity in MS patients while they perform predictable and unpredictable obstacle avoidance walking. The results showed that during unpredictable obstacle avoidance walking, healthy individuals exhibited lower cortical activation in the bilateral MC, indicating high neural processing efficiency. MS patients had lower cortical activation across most brain areas, suggesting potential limitations in neural resource allocation. When the tasks were combined, MS patients exhibited greater cortical activation than healthy controls did, indicating the involvement of compensatory mechanisms for maintaining gait stability (Al-Shargie et al., 2024). These results indicate the potential of combining fNIRS-based cortical activation with ML as a biomarker for MS-related impairments in cognitive‒motor interactions.

In addition to assessing neural activity during motor tasks in MS patients as mentioned above, fNIRS can also be used to evaluate neural activity in patients with different subtypes and varying degrees of MS severity. Hernandez et al. (2025) used fNIRS to measure differences in PFC activation during single-task and dual-task walking in older adults with progressive and relapsing-remitting MS. The increase in activity in the PFC related to the task (i.e., from single-task to dual-task walking) was greater in individuals with progressive MS than in those with relapsing-remitting MS, indicating that the PFC of progressive MS patients requires more compensation during dual-task walking. This indicates that the neural efficiency of progressive MS patients is lower than that of relapsing–remitting MS patients. fNIRS was subsequently used to measure the activation levels of the PFC during walking in MS patients with different levels of disability. The higher the degree of disability was, the higher the PFC activation level, indicating that individuals with higher levels of disability require greater compensatory activation of the PFC. After repeated practice, the greater the degree of disability was, the lower the decrease in PFC activation levels, indicating that individuals with higher levels of disability experience a greater decline in adaptability (Hernandez et al., 2024). In fact, researchers have proposed that the degree of cognitive impairment in progressive MS patients is greater. Additionally, previous studies have reported that accelerated brain atrophy is associated with primary progressive MS or the transition from relapsing–remitting MS to secondary progressive MS (Rimkus et al., 2024; Krijnen et al., 2025). Therefore, the aforementioned fNIRS examination of changes in cognitive control in the PFC of progressive and relapsing–remitting MS patients and MS patients with different levels of disability can provide a better understanding of the changes in function and potential brain responses caused by different MS subtypes and different levels of disability. In future studies, longitudinal neuroimaging studies of MS individuals can also be conducted to monitor MS progression from the perspective of brain neural activity. It may even be possible to predict the progression of MS.

## Future Directions

### Technological innovations and data standardization in functional near-infrared spectroscopy

To achieve higher precision, fNIRS hardware must evolve along two axes. First, the spatial resolution should be improved. Most current systems use a 3 cm source–detector separation, yielding centimeter-level resolution that only distinguishes large functional areas, without the ability to reveal fine-grained within-region differences (Vanegas et al., 2022). Adopting diffuse optical tomography with high-density optode arrays and 3-D reconstruction algorithms should increase the resolution to 5–10 mm, while simultaneously suppressing superficial scalp-blood artifacts and increasing the signal-to-noise ratio of neuro-hemodynamic signals (Hu et al., 2020). Second, a shift from measuring relative Hb concentration changes to measuring absolute Hb concentrations is needed. Continuous-wave devices—today’s mainstream—only detect relative concentration changes that depend heavily on baseline stability and suffer from linear drift during long recordings (Anaya et al., 2023). As time-domain fNIRS matures and becomes commercially available, its depth-resolved data and absolute hemoglobin concentrations will provide more stable, neuro-specific perfusion indices, facilitate the discovery of disease-specific biomarkers, and enable true long-term monitoring (e.g., intensive care unit surveillance, DOC assessment, and neonatal bedside imaging) (Ortega-Martinez et al., 2023).

Currently, the processing steps for fNIRS data remain nonuniform; therefore, further standardization is needed in the future to enhance the comparability of research findings. Data-processing standardization should focus on two aspects.

First, there is currently no standardized, highly operational pipeline or algorithm for evaluating fNIRS data quality (Nguyen et al., 2021). Commonly used approaches include the following: (1) Physiological-noise-based criteria—Because fNIRS measures cerebral blood flow, the signal is contaminated by physiological noise (e.g., heart rate). A fast Fourier transform is applied, and the power spectrum is inspected; a clear peak at approximately 1 Hz is taken as evidence that the physiological signal has been captured. However, this method lacks quantitative thresholds, cannot rule out other artifacts, and confirms only the presence of physiological—not necessarily neurovascular—fluctuations; (2) Raw intensity criteria—Metrics such as the signal-to-noise ratio or absolute light intensity can be examined, but no universal cut-offs exist, making the procedure experience-dependent and difficult to standardize; and (3) Motion-artifact criteria—Since cerebral hemodynamics fluctuate slowly while motion produces abrupt shifts, algorithms such as the coefficient of variation have been proposed (Kinder et al., 2022). However, the coefficient of variation thresholds vary widely (15%–35%) across studies, hindering harmonization (Yucel et al., 2025). To overcome these shortcomings, future work should develop an integrated, quantitative data-quality model that combines multidimensional information to deliver objective quality verification. Second, data processing still suffers from inconsistent pipelines and parameter settings (Tucker et al., 2023). Although motion-artifact removal and physiological noise reduction have become core preprocessing steps for fNIRS, no consensus gold standard exists for the exact methods or their order. Taking filtering as an example, some studies apply bandpass filters, whereas others use only low-pass filtering, since cerebral hemodynamic changes are predominantly low-frequency while physiological noise is higher (e.g., approximately heart rate 1 Hz, respiration 0.25 Hz) (Gervain et al., 2023). Although such method/parameter choices are often study-specific, future guidelines should unify standards and explicitly link research design, objectives, and processing approaches to establish a standardized pipeline with reference parameters.

### Future trends in the integration of functional near-infrared spectroscopy with artificial intelligence and its fusion into multimodal imaging

Artificial intelligence is currently advancing at breakneck speed. In the context of synergistic innovation between fNIRS and artificial intelligence (Andreu-Perez et al., 2021), this section discusses the core applications of deep learning algorithms in processing fNIRS brain function data. At the preprocessing stage, convolutional neural networks and related architectures achieve precise removal of motion artifacts and physiological noise. During feature mining, models are leveraged to extract hemodynamic signatures that are strongly coupled to brain functional states. Finally, at the predictive analytics stage, deep learning models enable real-time forecasting of dynamic oxygenation signals (Varandas et al., 2022).

Multimodal device integration is gradually becoming the mainstream future trend. As an optical imaging tool, fNIRS imposes minimal demands on the electromagnetic environment and thus offers excellent multimodal compatibility. The combination of EEG and fNIRS exploits the strengths of both techniques, simultaneously capturing millisecond-level neural activity and its spatial location (Ali et al., 2024). For example, high temporal-resolution EEG signals can be used to constrain the hemodynamic response function model used in fNIRS general linear model analyses, yielding individualized parameters. At the same time, fNIRS-derived spatial information can localize the specific cortical sources of event-related potential. Such coupling also provides a window for studying neurovascular coupling (Chen et al., 2023). Al-Shargie et al. (2017) reported that fusing EEG and fNIRS signals improved the accuracy of mental-stress detection. Beyond integration with other brain imaging modalities, pairing fNIRS with neuromodulation techniques such as TMS or tDCS also holds great promise (Saway et al., 2024). Synchronous real-time stimulation and functional monitoring can be used to directly visualize neuromodulatory effects and clarify the underlying mechanisms (Ghafoor et al., 2022). Of course, multimodal approaches still face challenges, including data integration and analysis methods; future work should focus on designing more fully integrated, all-in-one multimodal devices to facilitate clinical implementation. The NIR light employed by fNIRS results in a favorable signal-to-noise ratio, deep tissue penetration, and photothermal effects, making it suitable for specific phototherapeutic strategies, including photodynamic and photothermal therapies (Qin et al., 2022). By designing target-specific fluorescent probes, laser irradiation can selectively deliver significantly greater phototoxicity to abnormal lesions than to surrounding normal tissues under NIR imaging guidance, thereby achieving therapeutic goals (Wang et al., 2023b). Currently, the therapeutic potential of NIR photothermal effects for neurological disorders is under investigation. Yin et al. (2024) developed a polydopamine-modified black phosphorus nanosheet drug delivery system that exploits the NIR photothermal effect to enhance blood–brain barrier permeability and enable on-demand drug release. In a mouse model of middle cerebral artery occlusion, this platform markedly reduced infarct volume, alleviated cerebral edema, improved neurological deficits, and suppressed apoptosis and inflammatory responses, offering a novel strategy for treating ischemic stroke (Yin et al., 2024). Du et al. (2022) developed a niobium-carbide nanozyme that leverages the photothermal effects of NIR-II to enhance blood-brain barrier permeability while simultaneously scavenging reactive oxygen species and chelating Cu²⁺ ions. This dual function suppresses Cu^2+^-mediated reactive oxygen species generation and Aβ aggregation, ameliorating cognitive deficits in an AD mouse model and offering new insights for treating neurodegenerative disorders (Du et al., 2022). Wang et al. (2021a, b) developed an NIR-triggered nanophotosynthetic biosystem that employs upconversion nanoparticles to convert NIR light into visible light, thereby driving the cyanobacterium Synechococcus elongatus to photosynthesize, produce oxygen, and consume CO_2_, protecting neurons from hypoxic damage. In a murine stroke model, this system significantly improved neurological function and promoted angiogenesis, offering an innovative therapeutic strategy for ischemic stroke. All of the above studies exploit the NIR photothermal effect to enhance blood–brain barrier permeability, enabling the precise delivery of drugs or therapeutic agents. In the future, disease-specific targeted probes could enable the precise therapy of pathological lesions—such as Aβ plaques in AD patients or peri-focal edema in stroke patients. By integrating photothermal therapy with photoacoustic imaging, real-time monitoring and precise treatment of brain lesions can be achieved.

### Future clinical translation pathways for functional near-infrared spectroscopy

At present, the key barrier to the clinical translation of fNIRS is insufficient reliability, arising mainly from three obstacles: (1) Data and algorithmic hurdles: fNIRS signals have a low signal-to-noise ratio and are easily corrupted by head motion and ambient light. High-quality annotations of brain function data depend on expert personnel, while publicly available datasets, especially disease-specific fNIRS databases, remain scarce. Moreover, most current algorithms are trained on specific populations and perform poorly on children, elderly individuals, and other groups with pronounced interindividual differences. Additionally, these models lack robustness across centers and devices. (2) Standardization gaps: The field lacks unified technical standards; light-source wavelengths, detector sensitivities, and optode separations vary across manufacturers. Data-collection protocols are inconsistent in terms of sampling rates, stimulation paradigms, and recording durations, hindering cross-validation. Furthermore, no consensus exists on clinical evaluation criteria such as diagnostic thresholds or efficacy metrics. (3) Interpretability limitations: The causal relationship between hemoglobin saturation changes (HbO₂ and HbR) and underlying neuronal activity is not fully understood and is complicated by phenomena such as neurovascular–metabolic decoupling. The “black-box” nature of AI models renders the results opaque, leaving clinicians uncertain about their reliability. Additionally, healthcare personnel, particularly in primary-care settings, receive little training in fNIRS data interpretation, which limits clinical acceptance. Building on this foundation, it is important to establish standardized procedures. Developing unified standard processes for fNIRS signal acquisition, data processing, and analysis can enhance the reproducibility and comparability of research results. This requires international cooperation and consensus to establish standardized databases and sharing platforms that promote data sharing and exchange. It is also essential to establish a national fNIRS data-sharing platform in collaboration with universities, hospitals, and industry partners; formulate data-quality-control standards; and drive the development of annotated datasets. Strengthening translational research between basic science and clinical application is crucial for promoting the clinical translation of fNIRS technology. For example, conducting preclinical studies and early-phase clinical trials can help validate the potential application value of fNIRS in neurological disorders. However, it is also vital to develop clinical application guidelines and standards for using fNIRS in neurological disorders, clarifying its indications, operational procedures, and precautions to enhance its clinical value. When promoting the development of fNIRS applications in the field of neurology, attention should be given to interdisciplinary cooperation and communication, including neuroscience, physics, engineering, computer science, and clinical medicine, to facilitate the exchange and integration of knowledge and technology.

## Current Status of Clinical Translation and Application

fNIRS demonstrates high reproducibility and stability, suggesting significant potential for personalized, noninvasive assessment of brain function (**Box 1**).

Box 1: Progress in the clinical translation of the studies of functional near-infrared spectroscopy (fNIRS) in neurological disorders

**(1) Global distribution of clinical trials**

To understand the current clinical translation of fNIRS for use in neurological disorders, we searched the International Clinical Trials Registry Platform (ICTRP), ClinicalTrials.gov, and the Chinese Clinical Trial Registry website using the keyword “fNIRS.” A total of approximately 400 records were retrieved, and after screening, 82 clinical projects were identified as related to neurological disorders (**Additional Table 5**). These studies began in 2012 and were primarily conducted in the United States, New Zealand, and France. The focus of these studies is mainly on assessing rehabilitation for traumatic brain injury and stroke. Most of the studies are phase II trials, demonstrating the safety and reliability of fNIRS in monitoring brain activity (Popa et al., 2023). The findings provide preliminary evidence for the application of fNIRS in the field of collaborative neuroregeneration.

Relevant research entered a period of rapid growth in 2019, particularly in China. The scope and depth of the studied diseases significantly expanded, extending from stroke rehabilitation to conditions such as post-stroke depression and post-stroke cognitive decline. Research on neurodegenerative diseases and epilepsy also notably increased. Subsequently, these studies began to apply fNIRS to neuromodulation techniques such as transcranial direct current stimulation, transcranial magnetic stimulation (TMS), and BCI (Klein et al., 2024). The synergistic advancement of fNIRS and neuromodulation technologies has provided evidence from a neuroimaging-mechanism perspective for neuroregeneration research. With the development of BCI technology, the combination of fNIRS and EEG for noninvasive BCI signal acquisition and decoding may have a more direct and profound impact on promoting neural regeneration (Chai et al., 2025).

In the past 5 years, as the application scenarios for fNIRS in China have continuously expanded, studies using fNIRS in traditional Chinese medicine fields, such as acupuncture and tuina, have gradually emerged. This represents a typical case of the integration of modern technology with traditional medicine. fNIRS can provide a scientific explanation of the neural mechanisms underlying the effects of traditional medicine in improving conditions such as paralysis in stroke patients (Fu et al., 2023).

**(2) Financial support from the National Institutes of Health (NIH)**

The NIH has been funding fNIRS-related work since 2003, with support increasing sharply after 2016. Projects span a wide spectrum of disorders—any study that uses fNIRS to measure brain activity or function has been eligible. Due to the technology's high portability, recent grants emphasize real-world applications, such as monitoring gait or providing real-time feedback during cognitive training, underscoring the unique and irreplaceable role fNIRS plays in these specialized settings (https://reporter.nih.gov/search/B7DDQ8y7lUOkdo8peZRJHQ/projects). Over the past five years, funding agencies in both China and Europe have begun to support the implementation of fNIRS-related research (https://kd.nsfc.cn/finalProjectInit, https://cordis.europa.eu). In these projects, fNIRS is used as a tool to measure the neural mechanisms underlying diseases or social behaviors. Among the hot topics are hyperscanning, measurements in infants under one year old, patients with disorders of consciousness, integration with other technologies, and assistance in individualized neuromodulation.

Patents related to fNIRS have increased year by year. Early filings primarily focused on device hardware, while later ones covered the application of fNIRS to brain function measurement across various diseases, data analysis, and combined analyses with EEG and BCI (https://pss-system.cponline.cnipa.gov.cn, https://ppubs.uspto.gov/pubwebapp/static/pages/ppubsbasic.html, and https://worldwide.espacenet.com). This indicates that fNIRS has broad application prospects, and the emergence of patents in the future will further promote standardized data acquisition and analysis procedures for fNIRS.

The number of clinical studies employing fNIRS has increased year by year. As a convenient and rapid neuroimaging modality, fNIRS demonstrates high reproducibility and stability, making it a valuable tool for assessing brain function in neuroregeneration research (Lee et al., 2025). It also shows great potential in fields such as neuromodulation and brain–computer interfaces (Mihara et al., 2021). Finally, in the era of big data and artificial intelligence, the multimodal fusion of fNIRS with other technologies facilitates personalized and precise assessments of brain function (Li et al., 2025).

## Limitations

Based on the above discussion, fNIRS has been widely applied in neurological disorders. However, several limitations still exist.

First, the technical principles and equipment for fNIRS are limited. Compared to fMRI, fNIRS has lower spatial resolution and cannot precisely locate neural activity in the cerebral cortex. The detection depth of fNIRS is also restricted; it generally can only detect neural activity up to 1.5–2 cm below the cortex. This limitation affects its application for diseases in some deep brain regions. Additionally, fNIRS signals are susceptible to interference from tissues such as the scalp and hair, while heartbeat and respiration can also introduce noise.

Second, there are limitations related to research methods and data processing. To date, standardized procedures for fNIRS signal acquisition and data processing have not been established, leading to poor reproducibility and comparability among fNIRS-related studies (Wu et al., 2021; Yucel et al., 2025). This lack of standardization hampers the application and promotion of fNIRS in clinical practice. Furthermore, the sample sizes of current studies are relatively small, and the issue of statistical power limits the credibility of the conclusions (Peng et al., 2024; Chu et al., 2025).

Finally, although we have repeatedly noted the advances in the integration of fNIRS with other technologies and in combination with neuromodulation techniques, many unresolved issues still remain. For example, in BCI systems for stroke rehabilitation, the ability to decode fNIRS signals is still unknown. These issues are still in the exploratory phase and lack mature guidelines and clinical application standards.

## Conclusion

How the anatomical architecture of the brain gives rise to complex functions remains an open question. The gap between structure and function spans not only the trajectory of macroscopic white matter fascicles but also the formation of functional circuits and the fine-tuned regulation of dynamic plasticity; no single scale or imaging modality can yet provide a complete answer. Numerous neuroimaging studies have therefore attempted to elucidate this relationship using EEG, MEG, fMRI, and PET (Fotiadis et al., 2024). These techniques have enriched our understanding from electrophysiological, hemodynamic, and molecular perspectives, yet the exploration of the brain is still in its infancy. Investigators have made substantial advances by employing functional imaging methods to clarify disease-related alterations in brain activity, identify reliable biomarkers and pathological targets, and evaluate treatment-induced functional changes (Hu et al., 2021). Owing to its high spatial resolution and whole-brain coverage, fMRI is the most widely used neurofunctional modality and profoundly influences the presurgical localization of epileptic foci, the prediction of motor recovery after stroke, and the mapping of network dysfunction in patients with neurodegenerative diseases. PET provides further insight into the pathological mechanisms underlying neurodegeneration, epilepsy, and brain tumors, serving as a platform for novel drug target discovery. EEG and MEG record the minute electrical potentials generated by large-scale neuronal synchrony, directly reflecting neuronal firing, and are extensively applied in seizure monitoring, seizure prediction, and real-time signal decoding for noninvasive BCIs (Gallois et al., 2022). However, all of these methods carry inherent limitations: fMRI imposes strict scanning conditions and demands high patient compliance; PET is costly and involves ionizing radiation; and EEG and MEG offer limited spatial resolution and are highly vulnerable to head motion artifacts. These shortcomings have motivated the search for a new imaging technique that can reconcile temporal and spatial resolution while being repeatedly usable in real-world environments. fNIRS, with its portability, lack of ionizing radiation, and robustness to motion artifacts, enables the real-time measurement of neural activity in naturalistic settings and offers a unique advantage for nerve regeneration research. In this review, we summarize advances in the use of fNIRS to assess stroke, PD, dementia, epilepsy, and various other neurological disorders. According to current research, fNIRS is mainly used to record changes in brain activity, better explore the pathogenesis of diseases, help predict the incidence of disorders, and facilitate the diagnosis of diseases. Additionally, fNIRS is expected to be utilized in conjunction with other techniques, such as BCIs, EEG, and tDCS, in the treatment of diseases. Existing studies have shown that fNIRS has a wide range of prospective clinical applications in the neurological field and merits further in-depth research in the future (Peng et al., 2024; Chu et al., 2025). Admittedly, current fNIRS applications are still dominated by cross-sectional, small-sample observational studies that exhibit considerable heterogeneity across protocols, and direct links between fNIRS metrics and nerve regeneration endpoints, such as synaptic plasticity, axonal outgrowth, or functional rewiring, remain to be established. This review systematically illustrates the potential of fNIRS for dynamically monitoring and promoting nerve regenerative plasticity from three interconnected perspectives: an in-depth exploration of neurovascular coupling mechanisms, the precise evaluation of neural activity, and technical innovation in therapeutic interventions. By integrating current applications of fNIRS with future translational research, this article builds a bridge from mechanistic studies to clinical nerve regeneration therapies. In addition, standardized fNIRS parameters applicable to a variety of neurological disorders are proposed; these broadly applicable guidelines offer clinicians and rehabilitation engineers a unified, reproducible, and scalable protocol, facilitating the integration of multicenter big data and the construction of large-scale models. This review also highlights the potential for synergistic applications of fNIRS with other modalities, underscoring the importance of multimodal integration. Such integration not only enhances spatial and temporal resolution but also provides multidimensional decision-support data for individualized and precision treatments. Future work should extend fNIRS from common disorders, such as stroke and PD, to rare genetic and metabolic diseases that are currently almost untouched. Their low prevalence and wide geographical distribution leave them lacking systematic functional imaging datasets, resulting in poorly understood pathogenesis and unidentified potential therapeutic targets. Owing to its high portability, low operating cost, and strong robustness to motion artifacts, fNIRS could replace some traditional diagnostic approaches that rely on large-scale equipment, ionizing radiation, or sedative drugs. However, large-scale, multicenter, longitudinal cohort studies are still needed to verify the strength of the associations between fNIRS metrics and molecular biomarkers, structural imaging, and clinical scales, as well as to establish AI-based standardized analysis pipelines and quality control criteria. With the rapid development of BCIs, intelligent rehabilitation robots, and other novel technologies, fNIRS can also serve as a high-quality auxiliary tool tightly coupled with these therapeutic modalities. This integration can create a closed-loop rehabilitation system capable of real-time monitoring, instantaneous feedback, and dynamic regulation, thereby maximizing nerve regenerative plasticity and functional recovery.

## Additional files:

***Additional Table 1:*** *Characteristics of fNIRS devices for neurological disease applications.*

***Additional Table 2:*** *Comparison of fNIRS and fMRI imaging techniques.*

***Additional Table 3:*** *Application of fNIRS in neuromodulation rehabilitation of stroke.*

***Additional Table 4:*** *Application of fNIRS in the exploration of new therapeutic techniques for patients with PD.*

***Additional Table 5:*** *Clinical trials of fNIRS applications in neurological disorders*

Additional Table 5 Clinical trials of fNIRS applications in neurological disorders

***Additional Figure 1:*** *Application of fNIRS in stroke.*

Additional Figure 1 Application of fNIRS in stroke.
fNIRS: Functional near-infrared spectroscopy; BCI: brain-computer interface; PISEI: postischemic stroke executive dysfunction; PSD: poststroke depression.

***Additional Figure 2:*** *Application of fNIRS in PD.*

Additional Figure 2 Application of fNIRS in PD.
fNIRS: Functional near-infrared spectroscopy; PD: Parkinson's disease; tDCS: transcranial direct current stimulation; OAB: overactive bladder; MRgFUS: magnetic resonance imaging-guided focused ultrasound surgery.

***Additional Figure 3:*** *Application of fNIRS in dementia and ALS.*

Additional Figure 3 Application of fNIRS in dementia and ALS.
AD: Alzheimer's disease; ALS: amyotrophic lateral sclerosis; BCI: brain.computer interface; fNIRS: functional near-infrared spectroscopy; FTD: frontotemporal dementia; MCI: mild cognitive impairment; MI: motor imagery.

***Additional Figure 4:*** *Application of fNIRS in epilepsy.*

Additional Figure 4 Application of fNIRS in epilepsy.
fNIRS: Functional near-infrared spectroscopy.

***Additional Figure 5:*** *Application of fNIRS in traumatic neurological disorders.*

Additional Figure 5 Application of fNIRS in traumatic neurological disorders.
CVR: Cerebrovascular reactivity; DOC: disorders of consciousness; fNIRS: functional near-infrared spectroscopy; SCI: spinal cord injury; TBI: traumatic brain injury; TCVI: traumatic cerebral vascular injury; tDCS: transcranial direct current stimulation;

***Additional Figure 6:*** *Application of fNIRS in other neurological disorders.*

Additional Figure 6 Application of fNIRS in other neurological disorders.
CGRP: Calcitonin gene-related peptide; DAVFs: dural arteriovenous fistulas; fNIRS: functional near-infrared spectroscopy; MS: multiple sclerosis; NHND: nonhemorrhagic neurological deficits.

## Data Availability

*All relevant data are within the paper and its Additional files.*
