# Supplementary material for: Functional near-infrared spectroscopy: Systematic mapping of abnormal brain function features in neurological disorders
Source: Neural Regen Res. 2026 Jan 2;21(10):4506–22. doi: 10.4103/NRR.NRR-D-25-00595 (PMC13568639; doi:10.4103/NRR.NRR-D-25-00595)
Supplement: Supplementary file 1 [file NRR-21-4506_Suppl1.pdf]

Additional Table 5 Clinical trials of fNIRS applications in neurological disorders

Researcher	y+B2:K 73ear	ID	Country	Intervention	Research population	Experimental design	Primary endpoint events	URL	Source	Recruitment status
**Amir Gandjbakhche**	2012	NCT01668758	United States	NA	Traumatic brain injury	Observational	The main outcome measures will be to identify if the brain flow hemodynamic between the healthy and the TBI group is affected by trauma.	https://clinicaltrials.gov/show/NCT01668758	International Clinical Trials Registry Platform	Withdrawn
**Ramon Diaz-Arrastia**	2013	NCT01789164	United States	NA	Traumatic brain injury	Observational	Reproducibility of CVR measurements with fNIRS during intersession and intra-session testing (Time Frame: 2 yr)	https://clinicaltrials.gov/show/NCT01789164	International Clinical Trials Registry Platform	Completed
**Uniformed Services University of the Health Sciences**	2013	NCT01797549	United States	NA	Traumatic brain injury	Observational	Measure of hemodynamic response function (Time Frame: 2 yr)	https://clinicaltrials.gov/show/NCT01797549	International Clinical Trials Registry Platform	Completed
**Ramon Diaz-Arrastia**	2016	NCT02990078	United States	Drug: Sildenafil citrate; Other: Hypercapnia challenge; Device: fNIRS	Traumatic brain injury	Observational	Change in CVR prior to sildenafil administration (Time Frame: < 72 h, 14 d, 90 d, 180 d, 1 yr, 2 yr, 3 yr, 4 yr)	https://clinicaltrials.gov/show/NCT02990078	International Clinical Trials Registry Platform	Terminated
**Toshiyasu Ogata**	2016	JPRN-UMIN000021500	Japan	fNIRS and high-resolution volumetric T1-weighted imaging will be performed twice, 2 wk and 6 mon after stroke	Stroke	Single arm non-randomized	The primary outcome is to elucidate whether cognitive impairment may occur 6 months after a stroke in patients with cerebellar involvement.	https://center6.umin.ac.jp/cgi-open-bin/ctr_e/ctr_view.cgi?recptno=R000024777	International Clinical Trials Registry Platform	Completed
**Jérôme Froger**	2016	NCT02664636	France	Procedure: Occupational therapy	Stroke	Endpoint Classific	Fugl-Meyer motricity score [Time Frame: Day 60] Near-infrared spectroscopy	https://clinicaltrials.gov/show/	International Clinical Trials	Completed

	Procedure: Functional near-infrared spectroscopy<br>Procedure: Physiotherapy	ation: Efficacy Study, Intervention Model: Single Group Assignment, Masking: Open Label, Primary Purpose: Treatment	signal analysis: the amplitude peak [Time Frame: Day 0]<br>Near-infrared spectroscopy signal analysis: the average amplitude of cortical activation [Time Frame: Day 60]<br>Near-infrared spectroscopy signal analysis: the laterality index [Time Frame: Day 45]<br>Near-infrared spectroscopy signal analysis: the time required to reach the peak [Time Frame: Day 15]<br>Voluntary force: average [Time Frame: Day 45]<br>Voluntary force: the target error which measures the difference between the average of the force developed by the arm and target strength [Time Frame: Day 30]<br>Fugl-Meyer motricity score [Time Frame: Day 0]<br>Near-infrared spectroscopy signal analysis: the amplitude peak [Time Frame: Day 45]<br>Near-infrared spectroscopy signal analysis: the amplitude peak [Time Frame: Day 60]<br>Near-infrared spectroscopy	NCT02664636	Registry Platform

		signal analysis: the average amplitude of cortical activation [Time Frame: Day 0]<br>Near-infrared spectroscopy signal analysis: the time required to reach the peak [Time Frame: Day 0]<br>Fugl-Meyer motricity score [Time Frame: Day 30]<br>Near-infrared spectroscopy signal analysis: the average amplitude of cortical activation [Time Frame: Day 45]<br>Voluntary force: average [Time Frame: Day 60]<br>Voluntary force: the target error which measures the difference between the average of the force developed by the arm and target strength [Time Frame: Day 45]<br>Near-infrared spectroscopy signal analysis: the laterality index [Time Frame: Day 0]<br>Voluntary force: the target error which measures the difference between the average of the force developed by the arm and target strength [Time Frame: Day 60]<br>Near-infrared spectroscopy	

signal analysis: the time required to reach the peak [Time Frame: Day 45]
Voluntary force: average [Time Frame: Day 15]
Voluntary force: coefficient of variation [Time Frame: Day 0]
Voluntary force: coefficient of variation [Time Frame: Day 60]
Voluntary force: coefficient of variation [Time Frame: Day 30]
Fugl-Meyer motricity score [Time Frame: Day 15]
Near-infrared spectroscopy signal analysis: the amplitude peak [Time Frame: Day 30]
Voluntary force: average [Time Frame: Day 30]
Voluntary force: the target error which measures the difference between the average of the force developed by the arm and target strength [Time Frame: Day 15]
Near-infrared spectroscopy signal analysis: the laterality index [Time Frame: Day 15]
Near-infrared spectroscopy signal analysis: the laterality index [Time

		Frame: Day 60]<br>Near-infrared spectroscopy signal analysis: the time required to reach the peak [Time Frame: Day 60]<br>Voluntary force: average [Time Frame: Day 0]<br>Voluntary force: coefficient of variation [Time Frame: Day 15]<br>Voluntary force: the target error which measures the difference between the average of the force developed by the arm and target strength [Time Frame: Day 0]<br>Fugl-Meyer motricity score [Time Frame: Day 45]<br>Near-infrared spectroscopy signal analysis: the amplitude peak [Time Frame: Day 15]<br>Near-infrared spectroscopy signal analysis: the average amplitude of cortical activation [Time Frame: Day 15]<br>Near-infrared spectroscopy signal analysis: the average amplitude of cortical activation [Time Frame: Day 30]<br>Near-infrared spectroscopy signal analysis: the laterality index [Time	

							Frame: Day 30]<br>Near-infrared spectroscopy signal analysis: the time required to reach the peak [Time Frame: Day 30]<br>Voluntary force: coefficient of variation [Time Frame: Day 45]			
**An na LEE**	2018	KCT0003257	Korea	Robot group: 1 hour of conventional rehabilitation treatment + Morning work therapy 30 minutes<br>- Control group: Conventional rehabilitation treatment 1 hour and 30 minutes	Stroke	Primary Purpose: Treatment, Intervention Model: Parallel, Blinding/Masking: Open, Allocation: RCT	Functional near-infrared spectroscopy	http://cris.nih.go.kr/cris/en/search/search_result_st01.jsp?seq=15731	International Clinical Trials Registry Platform	Completed
**University Hospital, Limoges**	2018	NCT03559283	France	Diagnostic Test: Walking and Record Cortical Activity	Stroke	Allocation: N/A. Intervention model: Single Group Assignment. Primary purpose: Diagnostic. Masking	Cost of the Double Task [Time Frame: Day of the Inclusion]	https://clinicaltrials.gov/show/NCT03559283	International Clinical Trials Registry Platform	Completed

						: None (Open Label).				
**Ni Jing**	2019	ChiCTR 1900028 495	China	Experimental group: using repetitive transcranial magnetic stimulation; Control group: using functional near infrared spectroscopy;	Stroke	Parallel	Fugl-Meyer movement function rating scale; functional near infrared spectroscopy;	http://www.chictr.org.cn/showproj.aspx?proj=41614	International Clinical Trials Registry Platform	Completed
**Minho Chun**	2019	KCT000 4381	Korea	Medical Device: Robot group: Gait Rehabilitation treatment using Healbot G, 30 minutes; Control group: Gait rehabilitation treatment using Treadmill, 30 minutes	Stroke	Primary Purpose: Treatment, Intervention Model: Parallel, Blinding /Masking: Open, Allocation: RCT	Functional near-infrared spectroscopy	http://cris.nih.go.kr/cris/en/search/search_result_st01.jsp?seq=17468	International Clinical Trials Registry Platform	Completed
**Radboud Universiteit Nijmegen**	2019	NL-OM ON4947 9	Netherlands	NA	Parkinson's disease	Open (masking not used), Uncontrolled, Basic science	The main study parameter is change in fNIRS activity (i.e., oxygenated hemoglobin and de-oxygenated hemoglobin) relative to baseline in 10 brain regions: the sensorimotor cortex, the supplementary motor area,	https://onderzoekmetmensen.nl/en/trial/49479	International Clinical Trials Registry Platform	Completed

							the premotor cortex, the dorsolateral prefrontal cortex, and the posterior parietal (in both hemispheres).			
**Kimbra Kenney**	2019	NCT04058132	United States	Other: Neuropsychological assessments; Other: Structural brain magnetic resonance imaging (MRI; Drug: Gadolinium contrast infusion; Procedure: Blood sample collection for research purposes; Device: functional near-infrared spectroscopy; Drug: Sildenafil Citrate 50Mg Tab; Other: Carbon Dioxide (CO_2) 5%	Traumatic brain injury	Allocation: Non-Randomized. Intervention model: Parallel Assignment. Primary purpose: Basic Science. Masking: None (Open Label).	Detection of variation of oxyhemoglobin and deoxyhemoglobin concentration using a power analysis between groups during the hypercapnia challenge pre and post a single dose of sildenafil 50 mg at the specified time points after a TBI. Longitudinal measure of CVR between groups [Time Frame: 2 years]	https://clinicaltrials.gov/ct2/show/NCT04058132	International Clinical Trials Registry Platform	Terminated
**Nam-Jong Paik**	2019	NCT04288414	Korea	Diagnostic Test: functional near-infrared spectroscopy	Stroke	Observational	Change of Outcome Prediction Rate [Time Frame: After 2-3 wk from stroke onset (T0), Immediately after 2-3 wk of rehabilitation (T1), After 1-3 months from stroke onset (T2)]	https://clinicaltrials.gov/ct2/show/NCT04288414	International Clinical Trials Registry Platform	Recruiting
**Yifang Lin**	2020	ChiCTR2000036	China	Electroacupuncture the acupoint	Stroke	Single arm	Fugl-Meyer motor assessment;	http://www.chictr.o	International Clinical	Pending

		959		LI11, LI 10, LI4;				rg.cn/showproj.aspx?proj=60060	Trials Registry Platform	
**Jie Jia**	2020	ITMCTR2000003717	China	Electroacupuncture the acupoint LI11, LI 10, LI4;	Stroke	Single arm	Fugl-Meyer motor assessment;	http://itmctr.ccebtcm.org.cn/en-US/Home/ProjectView?pid=fb64dbfd-4922-4ec0-8aeb-d2f503544ac0	International Clinical Trials Registry Platform	Pending
**Huiyu Liu**	2020	ChiCTR2000032804	China	Patient group: tDCS; Healthy people group: tDCS;	Post-stroke cognitive impairment	Factorial	MMSE Scale; MoCA Scale;	http://www.chictr.org.cn/showproj.aspx?proj=53407	International Clinical Trials Registry Platform	Pending
**Kaiqi Huang**	2020	ChiCTR2000038588	China	Bionic electrical stimulation therapy + Routine rehabilitation program	Cognitive Impairment after stroke	Parallel	Changes in Montreal Cognitive Assessment Scale (MOCA) scores; Hamilton Anxiety Scale (HAMA); VFT	NA	Chinese Clinical Trial Registry	Prospective registration
**Ma Jinrong**	2020	ChiCTR2000031625	China	Thorn acupuncture and medication	Cognitive Impairment after stroke	Parallel	Hemoglobin content	NA	Chinese Clinical Trial Registry	Prospective registration
**Chen Yan**	2021	ChiCTR2100053496	China	Experimental group: Upper limb rehabilitation robot; control group: Routine rehabilitation therapy	stroke	Parallel	the upper-extremity portion of the Fugl-Meyer motor assessment; Action Research Arm Test; modified Barthel index; TMS related MEP indicators; FNIRS	http://www.chictr.org.cn/showproj.aspx?proj=134293	International Clinical Trials Registry Platform	Recruiting

							indicators;			
**Canan OZSANCAK**	2021	NCT05073874	France	Other: Functional near-infrared spectroscopy	Parkinson's disease	Allocation: N/A. Intervention model: Single Group Assignment. Primary purpose: Basic Science. Masking: None (Open Label).	Effects of deoxyhemoglobin variations on hemodynamic response in prefrontal cortex;<br>Effects of oxyhemoglobin variations on hemodynamic response in prefrontal cortex [Time Frame: Baseline]	https://clinicaltrials.gov/ct2/show/NCT05073874	International Clinical Trials Registry Platform	Completed
**Huo Congcong**	2021	ChiCTR2100051148	China	Group 1: Multi-target repetitive transcranial magnetic stimulation (rTMS); Group 2: Multi-target rTMS combined with limb movement rehabilitation training; Group 3: Multi-target functional electrical stimulation (FES); Group 4:	stroke	Parallel	Cerebral oxygenation;<br>Clinical functional scale;	http://www.chictr.org.cn/showproj.aspx?proj=133870	International Clinical Trials Registry Platform	Recruiting

				Multi-target FES combined with limb movement rehabilitation training;						
**Sheng Yilan**	2021	ChiCTR 2100049 190	China	Experimental group: electroencephalograph bionic therapy; Control group: electroencephalograph bionic sham therapy;	stroke	Parallel	N-back Task; Near infrared test	http://www.chictr.org.cn/showproj.aspx?proj=130856	International Clinical Trials Registry Platform	Pending
**Ma Xiancong**	2021	ChiCTR 2100048 914	China	Experimental group: repetitive transcranial magnetic; Control group: fake repeat transcranial magnetic;	Post-stroke cognitive impairment	Parallel	Brief Mental State Inventory; Montreal Cognitive Assessment Scale;	http://www.chictr.org.cn/showproj.aspx?proj=130228	International Clinical Trials Registry Platform	Pending
**Shen Ying**	2021	ChiCTR 2100048 807	China	Experimental group: contralaterally controlled functional electrical stimulation; control group: neuromuscular electrical stimulation	Stroke	Parallel	Functional near-infrared spectroscopy	http://www.chictr.org.cn/showproj.aspx?proj=129634	International Clinical Trials Registry Platform	Pending
**Ma Xiancong**	2021	ITMCTR21000 05051	China	Acupuncture treatment and routine swallowing	Dysphagia after stroke	Parallel	Rosenbek osmotic aspiration score; GUSS swallowing function assessment form; fiberoptic	http://itmctr.ccebtcm.org.cn/en-US/Hom	International Clinical Trials Registry	Recruiting

				therapy: Acupuncture Treatment; Transcranial direct current stimulation and routine swallowing therapy group: tDCS treatment; control group: routine swallowing therapy;			endoscopic dysphagia severity scale; standard swallowing function assessment scale; swallow assessment scale; swallow severity scale;	e/ProjectView?pid=35b5c16a-0eec-4a68-805c-37568e26ae8e	Platform	
**Peng Yang**	2021	ChiCTR2100048703	China	Acupuncture treatment and routine swallowing therapy: acupuncture treatment; transcranial direct current stimulation and routine swallowing therapy group: tDCS Treatment; control group: Routine swallowing therapy	dysphagia after stroke	Parallel	Standard Swallowing Function Assessment Scale; Fiberoptic Endoscopic Dysphagia Severity Scale; Swallow severity scale; GUSS swallowing function assessment form; Rosenbek osmotic aspiration score;	http://www.chictr.org.cn/showproj.aspx?proj=130225	International Clinical Trials Registry Platform	Recruiting
**Thao TP Nguyen**	2021	NCT04941482	Vietnam	Behavioral: Guiding the appropriate rehabilitation	Stroke	Allocation: Randomized.	Changes from Baseline Patient Health Questionnaire (PHQ-9) at 1, 3, and 6 months;	https://clinicaltrials.gov/ct2/show/NCT04	International Clinical Trials Registry	Completed

				exercises Behavioral: Periodic health check program for post-stroke Behavioral: Motivational Interviewing Device: Functional near-infrared spectroscopy		Intervention model: Parallel Assignment. Primary purpose: Prevention. Masking: Single (Outcomes Assessor).	Changes from Baseline Barthel Index (BI) at 1, 3, and 6 months; Changes from Baseline Mini-Mental State Examination (MMSE) at 1, 3, and 6 months; Changes from Baseline Stroke Impact Scale (SIS) at 1, 3, and 6 months [Time Frame: Baseline, 1, 3, and 6 months post intervention]	941482	Platform	
**Ran An**	2021	ChiCTR2100043288	China	Experimental group: unstable support surface training; control group: Nil	Parkinson's disease	Parallel	Berg Balance Scale Scale; Changes in cortical activation	http://www.chictr.org.cn/showproj.aspx?proj=121821	International Clinical Trials Registry Platform,ICTRP	Recruiting
**Kim-Charline Broscheid**	2021	DRKS00022342	Germany	Group 1: Stroke patients: The participants are informed in advance about the study procedure and written consent is obtained. A 6-minute walking test is performed before the actual measurement date. The main	Ischemic Stroke	Allocation: Masking; Study design purpose: basic science	Oxy- and deoxyhemoglobin concentrations in the prefrontal cortex (functional near-infrared spectroscopy); double step length as well as minimum foot clearance and the variability (three inertial measurement Units: two dorsally fixed at each foot and one at the Sternum)	http://drks.de/search/en/trial/DRKS00022342	International Clinical Trials Registry Platform	Completed

measurements are taken on two consecutive test days (24 hours in between) on non-treatment days at the same time of the day. The same procedure is performed on both days to determine the test-retest reliability. At the beginning of the first test, the participants are given questionnaires (Beck-Depressions-Inventory Revision, Fatigue Severity Scale), which they should bring along on the retest day. In addition, a color-word interference test will be conducted in printed form. Afterwards, the measuring equipment (functional near-infrared

spectroscopy, inertial sensors on the feet and sternum, 3-channel ECG and a mouse fixed in the hand) is put on.
The subjects then completed two different walking protocols. The first walking protocol lasted a total of 4:30 min and the participants changed every 30 seconds between standing (baseline) and walking at a comfortable speed. They began and ended with a baseline (standing). The second walking protocol was 6 minutes long in total. Here, they started and ended also with a baseline (standing) and in between they

				walk for 2 minutes. While walking, the participants reacted to acoustic stimuli by clicking the mouse fixed in the dominant hand. Two different tones were played to the test person in random order and they only reacted to the lower tone. Both walking protocols were performed in random order. Group 2: Healthy controls (see stroke patients)						
**Northumbria University**	2021	NCT04863560	United States and United Kingdom	Auditory Cueing; Visual Cueing; tactile cueing	Parkinson's disease	Observational	Change in cortical oxygenated hemoglobin (HbO_2) signal during walking [Time Frame: immediately after intervention] Change in cortical power spectral densities during walking [Time Frame: immediately after intervention]	NA	Clinical Trials	Unknown status
**University of Sao**	2021	NCT025	Brazil	tDCS and	Spinal	Allocati	Change in the Walk Index	NA	Clinical	Comple

**Paulo General Hospital**		62001		Lokomat Training	cord injury	on Randomized Interventional Model Parallel Assignment Masking Double (Participant Investigator) Primary Purpose Treatment	for Spinal Cord Injury, WISCI II [Time Frame: pre (before treatment) [t0], inter (after 15 sessions) [after 5 weeks GA and after 3 weeks GI], post (after treatment) [after 10 weeks GA and after 6 weeks GI] and 3 months follow up [after 22 weeks GA and after 18 weeks GI]]		Trials	ted
**Zhang Qiaojun**	2021	ChiCTR2100052689	China	NA	Traumatic brain injury	Factorial	Resting brain function connection;<br>Verbal fluency test, VFT	NA	Chinese Clinical Trial Registry	Prospective registration
**Zhang Qiaojun**	2021	ChiCTR2100052623	China	Transcranial direct current stimulation + Routine rehabilitation program	Stroke	Parallel	Verbal Fluency Test;<br>Working Memory;<br>Go/No-Go	NA	Chinese Clinical Trial Registry	Prospective registration
**Xiaoman Liu**	2021	ChiCTR2100049921	China	acupuncture	Post-stroke depression	Single arm	Peak/average value of oxygenated hemoglobin concentration during the task stage (peak oxy-Hb (mM·mm) / average oxy-Hb (mM·mm)); initial activation speed of	NA	Chinese Clinical Trial Registry	Retrospective registration

							oxygenated hemoglobin during the task stage (K-activation); the integral value of the activation response of oxygenated hemoglobin during the task stage (S-integration);the reaction time when the integral value of the oxygenated hemoglobin reaches half of that in the whole process (T-center)			
**Liu Yong**	2022	ChiCTR 2200066 236	China	Control group: Regular training; Cognitive intervention group: Regular training + Cognitive intervention training; rTMS group: Regular training + rTMS stimulation; Combined group: Regular training + Cognitive intervention training + rTMS stimulation;	Cognitive disorder	Parallel	Mini-mental state examination, MMSE;	https://www.chictr.org.cn/showproj.html?proj=186611	International Clinical Trials Registry Platform	Recruiting
**Yang Tian**	2022	ChiCTR 2200065 838	China	AOT1: action observation; AOT2: action observation plus imagination; AOT3: action	stroke	Cross-over	HbO2;	https://www.chictr.org.cn/showproj.html?proj=185580	International Clinical Trials Registry Platform,ICTRP	Pending

				observation plus execution; AOT4: action observation plus imagination and execution; MT1: mirror therapy; MT2: mirror therapy plus imagination; MT3: mirror therapy plus execution; MT4: mirror therapy plus imagination and execution						
**Lihua Nong**	2022	ChiCTR2200065522	China	Trial group: A single extracorporeal shock wave was applied to the patient's hamstring, gastrocnemius, and soleus muscles; Control group: Sham stimulation was provided by playing the ESW treatment sound without turning the device on in contact with the skin; Healthy people group: A single	Spasticity in Hemiplegic Legs of Stroke Patients	Parallel	Functional brain imaging with near-infrared spectroscopy monitored oxygen-hemoglobin concentrations in bilateral primary sensorimotor cortex; Modified Ashworth Scale (MAS); A-ROM?P-ROM; Brunnstrom Rating Scales;	https://www.chictr.org.cn/showproj.html?proj=183203	International Clinical Trials Registry Platform	Pending

				extracorporeal shock wave was applied to the patient's hamstring, gastrocnemius, and soleus muscles; Primary Outcome(s)						
**Qian Zhang**	2022	ChiCTR 2200065 153	China	Conventional treatment: conventional swallowing training; intermittent theta burst stimulation; repetitive transcranial magnetic stimulation	Stroke	Parallel	VFSS quantitative analysis: Movement distance of hyoid lift and advance; MBSImP scale; surface electromyography: Mean values of swallowing duration and maximum amplitude of bilateral suprhyoid muscle groups; Motor evoked potential; Mean HbO of bilateral IGF region, SMC region and SMA region	https://www.chictr.org.cn/showproj.html?proj=184263	International Clinical Trials Registry Platform,ICTRP	Pending
**Yongfeng Hong**	2022	ChiCTR 2200063 792	China	Group 1: Detection of fMRI and fNIRS; Group 2: Giving regular rehabilitation treatment; Group 3: Give regular rehabilitation treatment and MDSS treatment;	Stroke	Parallel	Modified Ashworth Myospasm Scale (MAS); Manual muscle strength testing (MMT); Fugl-Meyer assessment of overall hand and upper extremity function; Modified Barthel Index, MBI; root mean square value of surface electromyography; blood oxygen concentration	https://www.chictr.org.cn/showproj.html?proj=176575	International Clinical Trials Registry Platform	Pending
**Xia Li**	2022	ChiCTR 2200059	China	Experiment group:2	Post-stroke	Parallel	fNIRS	https://www.chictr.o	International Clinical	Pending

		702		psychological counseling per week for 30 minutes each time. And they were instructed to light a stick of incense made of Amomum tsaoko Crevost et Lemaire and Aquilaria sinensis Lour every day; Control group:2 psychological counseling per week for 30 minutes each time;	depression			rg.cn/showproj.html?proj=167303	Trials Registry Platform	
**Wan Chunxiao**	2022	ChiCTR2200059189	China	Case group: Repeat transcranial magnetic stimulation; Control group: Repeat transcranial magnetic stimulation	Spinal cord disease	Cross-sectional	ASIA Motor Function Score; ASIA Sensory Score;	https://www.chictr.org.cn/showproj.html?proj=166698	International Clinical Trials Registry Platform	Pending
**Songmei Chen**	2022	ChiCTR2200057449	China	Group A: True Stimulation; Group B: Shame Stimulation;	Post-stroke cognitive impairment	Parallel	Mini-Mental State Examination	https://www.chictr.org.cn/showproj.html?proj=162868	International Clinical Trials Registry Platform	Recruiting
**Min Ho Chun**	2022	KCT000	Korea	Experimental	stroke	RCT	fNIRS	https://cris	International	Comple

		7053		group: Upper limb rehabilitation training with rebless 30 minutes; Control group: Upper limb rehabilitation training with palliative rehabilitation (e.g., motomed) 30 min				.nih.go.kr/cris/search/detailSearchEn.do?seq=22727	Clinical Trials Registry Platform	ted
**Bin Ye**	2022	ITMCTR2200005595	China	TMS and TMS group: TMS and TMS; TuiNa group: TuiNa; TMS group: TMS	stroke	Parallel	Fugl-meyer motor function score of the affected upper limb;	http://itmctr.ccebtcm.org.cn/en-US/Home/ProjectView?pid=bb988d04-d25d-4510-a8de-a5458987e4d6	International Clinical Trials Registry Platform	Pending
**David Lin**	2022	NCT05258591	United States	Device: Axem Home	Hemiparesis after stroke	Allocation: N/A. Intervention model: Single Group Assignment. Primary purpose: Treatme	Determine when during the study (if at all) fNIRS data contains predictive information regarding the likelihood that participants would respond to at-home BCI rehabilitation. Determine whether there are participants who respond to at-home upper-extremity BCI rehabilitation. [Time Frame:	https://clinicaltrials.gov/show/NCT05258591	International Clinical Trials Registry Platform	Recruiting

						nt. Masking : None (Open Label).	6 weeks]			
**Seoul National University Bundang Hospital**	2022	NCT02591719	Korea	Device: MagPro magnetic stimulator (HF rTMS); Device: MagPro magnetic stimulator (sham) ; Device: MagPro magnetic stimulator (LF rTMS)	Aphasia of Stroke	Allocation Randomized Interventional Model Parallel Assignment Masking Quadruple (Participant Care Provider Investigator Outcomes Assessor) Primary Purpose Treatment	BNT (Boston Naming Test) [Time Frame: up to 2 weeks]	NA	Clinical Trials	Unknown status
**Seoul National University Bundang Hospital**	2022	NCT04290377	Korea	BMI-assisted motor imagery OT; Motor imagery OT	Stroke Patients with Hemiplegia	Allocation Randomized Interven	Change of Fugl Meyer Assessment-Upper Limb (FMA-UL) Score [Time Frame: Baseline evaluation (T0), Evaluation after 2	NA	Clinical Trials	Unknown status

			tional Model Parallel Assignment Interventional Model Description: Phase1 (chronic stroke): with-in group (one group) multiple measurements Phase2 (subacute stroke): Randomized controlled study. Control (OT plus OT) vs Experimental (OT	weeks of therapy (T1 [immediately or 1 day after treatment termination]), Evaluation after 1 month of T1 (T2), Evaluation after 3 mon of T2 (T3).]

						plus BMI-assisted OT) Masking Double (Investigator Outcomes Assessor) Masking Description: Impossible to mask participants and care providers due to using a device that is so obvious. Primary Purpose Treatment				
**Yufeng Chen**	2022	ChiCTR2200056266	China	TuiNa; TMS; TuiNa+TMS;	Ischemic Stroke	Parallel	Fugl-meyer motor function score of the affected upper limb	NA	Chinese Clinical Trial Registry	Prospective registration

**Li Fang**	2022	ChiCTR2200055413	China	Transcranial Magnetic Stimulation	stroke	Parallel	Fiberoptic Endoscopic Dysphagia Severity Scale; Penetration/Aspiration Scale; Gugging Swallowing Screen; Functional Oral Intake Scale; Standardized Swallowing Assessment; fNIRS	NA	Chinese Clinical Trial Registry	Prospective registration
**Marco Yiu Chung PANG**	2023	NCT06129227	Hong Kong	Behavioral: Control group Behavioral: Dual-task training Behavioral: Single-task training	Stroke	Allocation: Randomized. Intervention model: Parallel Assignment. Primary purpose: Treatment. Masking: Single (Outcomes Assessor).	Dual-task gait speed; Dual-task cognitive performance [Time Frame: Through study completion, an average of 1 year]	https://clinicaltrials.gov/ct2/show/NCT06129227	International Clinical Trials Registry Platform	Recruiting
**Jin Tao**	2023	ChiCTR2300075703	China	Conventional rehabilitation group: Placebo stimulation; Healthy side CTBS intervention group: CTBS	Stroke	Parallel	Resting functional near infrared spectroscopy;	https://www.chictr.org.cn/showproj.html?proj=205257	International Clinical Trials Registry Platform	Recruiting

				intervention on the healthy side M1 area; Bilateral stimulation group: After CTBS intervention on the healthy side M1 area, iTBS intervention on the affected side M1 area immediately						
**Dingqun Bai**	2023	NCT06062407	China	NA	Stroke	Observational	Preliminary screening of brain function biomarkers for fall risk in post-stroke patients;<br>The brain functional effectiveness of the EMG feedback-based standing training;<br>The neuromuscular activity of the EMG feedback-based standing training [Time Frame: 1 day]	https://clinicaltrials.gov/ct2/show/NCT06062407	International Clinical Trials Registry Platform	Recruiting
**Guanghua Liu**	2023	ChiCTR2300075378	China	Conventional cognitive training; Conventional cognitive training + music therapy	Stroke	Parallel	Montreal Cognitive Assessment, MoCA, MOCA; MMSE;	https://www.chictr.org.cn/showproj.html?proj=206322	International Clinical Trials Registry Platform	Recruiting
**Xia Wenguang**	2023	ChiCTR2300071569	China	NA	MCI and AD	Sequential	fNIRS	https://www.chictr.org.cn/showproj.html?proj=192644	International Clinical Trials Registry Platform	Pending

**Chen Xinxin**	2023	ChiCTR2300070734	China	NA	Epilepsy	Case-Control study	resting-state fNIRS	https://www.chictr.org.cn/showproj.html?proj=193898	International Clinical Trials Registry Platform,ICTRP	Pending
**Gong Yulai**	2023	ChiCTR2300069299	China	NA	Stroke	Case-Control study	Changes in hemoglobin concentration	https://www.chictr.org.cn/showproj.html?proj=188807	International Clinical Trials Registry Platform	Completed
**Zhiqi Mao**	2023	NCT06175897	China	Procedure: Deep Brain Stimulation; Procedure: non-Deep Brain Stimulation	Parkinson's Disease	Allocation Randomized Interventional Model Parallel Assignment Masking Double (Investigator Outcomes Assessor) Primary Purpose Treatment	Change in EEG-fNIRS performance [Time Frame: up to 1 month (DBS-on) ,6 months,12 months]	NA	Clinical Trials	Recruiting
**Chongqing Medical**	2023	NCT06094452	China	Computerized Cognitive	MCI and AD	Allocation	Global Cognition [Time Frame: baseline, 4, 12, 24	NA	Clinical Trials	Completed

**University**	Training	Randomized Interventional Model Parallel Assignment Interventional Model Description: After recruitment and baseline assessments, pairs of participants with MCI and mild AD will be randomly allocated into either the CCT group and the control group.	weeks]

Participants in CCT group will receive a multidomain adaptive computerized cognitive training program (www.66nao.com) for 24 wk. Patients in control group will receive treatment as usual (TAU) for 24 wk.
Masking Double (Investigator

	Outcomes Assessor) Masking Description: Since it will not be possible to blind the participants because the experimental and control interventions must be explained to them and their caregivers before randomization. The single blinding will be

						applied to researchers who conducted the measurements to minimize the potential assessor biases. Blinding will be also maintained for data management, outcome assessment, and data analysis. Primary Purpose Treatment				
**Song Jianfei**	2023	ChiCTR 2300076 155	China	Robot-assisted upper training rehabilitation + Conventional rehabilitation training	stroke	Parallel	fNIRS	NA	Chinese Clinical Trial Registry	Retrospective registration

**Dingqun Bai**	2024	NCT06703320	China	NA	Stroke Cognitive Decline	Observational	Brain Activation Pattern Caused by Immersive Virtual Reality Pathfinding Task in Stroke Patients [Time Frame: 1 day]		International Clinical Trials Registry Platform	Recruiting
**Fubiao Huang**	2024	ChiCTR2400090967	China	NA	Spinal cord injury	Cross-sectional	Montreal cognitive assessment	https://www.chictr.org.cn/showproj.html?proj=244295	International Clinical Trials Registry Platform	Completed
**Xuejing Li**	2024	ChiCTR2400090775	China	Health control: NONE; Acupuncture group: Acupuncture of Lianquan acupoint; Usual care group: Carry out basic rehabilitation training, with acupuncture point pseudo-acupuncture;	stroke	Parallel	ß value	https://www.chictr.org.cn/showproj.html?proj=236802	International Clinical Trials Registry Platform	Completed
**Zhang Xiaohui**	2024	NCT06471309	China	Device: Theta burst transcranial magnetic stimulation	Stroke	Allocation: Randomized. Intervention model: Parallel Assignment. Primary	functional near- infrared spectroscopy；Rosenbek Penetration-aspiration scale；Standardized Swallowing Assessment [Time Frame: The first evaluation was before the intervention and the second evaluation was after 2 weeks of treatment.]	https://clinicaltrials.gov/ct2/show/NCT06471309	International Clinical Trials Registry Platform	Enrolling by invitation

						purpose: Treatment. Masking: Double (Participant, Outcomes Assessor)				
**The First Affiliated Hospital of Zhengzhou University**	2024	NCT06464835	China	Device: Transcranial magnetic stimulation	Stroke	Allocation: Randomized. Intervention model: Parallel Assignment. Primary purpose: Treatment. Masking: Double (Participant, Outcomes Assessor).	functional near-infrared spectroscopy; The Yale Pharyngeal Residue Severity Rating Scale; Standardized Swallowing Assessment Scale [Time Frame: immediately before and immediately after the intervention]	https://clinicaltrials.gov/ct2/show/NCT06464835	International Clinical Trials Registry Platform	Enrolling by invitation
**Ning Keyi**	2024	ChiCTR2400084987	China	NA	Parkinson's disease	Sequential	Functional near-infared spectroscopy; HAMD; HAMA;	https://www.chictr.org.cn/sho	International Clinical Trials	Pending

								wproj.htm l?proj=21 3235	Registry Platform	
**Zhu Hongjun**	2024	ChiCTR 2400084 545	China	transcranial magnetic stimulation	Stroke	Cohort study	Total score of the upper limb part of the Fugl-Meyer scale;	https://www.chictr.org.cn/showproj.html?proj=228522	International Clinical Trials Registry Platform	Pending
**DILINUER MAIMAITIAILI**	2024	ChiCTR 2400081 207	China	Group A (Group TIS):The patient will receive temporal interference stimulation; Group B (Group tACS): The patient will receive transcranial alternating current stimulation treatment	Stroke	Parallel	Montreal Cognitive Assessment, MoCA;	https://www.chictr.org.cn/showproj.html?proj=218273	International Clinical Trials Registry Platform	Pending
**Kernel**	2024	NCT059 96575	United States	fNIRS measurement	Mild Cognitive Impairment	Observational	Brain hemodynamic activity with TD-fNIRS Optical properties of the brain with TD-fNIRS Physiological features with TD-fNIRS [Time Frame: About 1 hour during the study visit]	NA	Clinical Trials	Completed
**Karolinska Institutet**	2024	NCT052 18213	Sweden	NA	Parkinson's Disease	Observational	Functional near infrared spectrometry [Time Frame: During the test session during all three conditions] Gait performance during all	NA	Clinical Trials	Completed

							conditions [Time Frame: During the test session during all three conditions] Dual-task performance-reaction time [Time Frame: During the test session during dual task conditions]			
**University of Calgary**	2024	NCT05016193	Canada	Device: Axem Home Prototype Device; Behavioral: Rehabilitation Exercise Program	Stroke	llocation Randomized Interventional Model Parallel Assignment Masking Single (Outcomes Assessor) Primary Purpose Other	Incidence of Adverse Events Related to the Axem Home Prototype Device (Safety and Tolerability) [Time Frame: Intervention group only: through study completion, an average of 6 weeks] Usability Interview [Time Frame: Intervention group only: once per week for an average of 6 weeks and post-intervention (after study completion, an average of week 6)] Usability Questionnaire [Time Frame: Intervention group only: post-intervention (after study completion, an average of week 6)] Participation [Time Frame: Intervention group only: through study completion, an average of 6 weeks]	NA	Clinical Trials	Completed
**Shanshan Zhou**	2024	ChiCTR2400093089	China	NA	Post-stroke depression	Cohort study	Prefrontal cortex oximetry;Hamilton Depression Scale (17-item)	NA	Chinese Clinical Trial	Prospective registrat

							scores		Registry	ion
**Yalu Sun**	2024	ChiCTR 2400088 351	China	Virtual reality training and traditional rehabilitation training	Ischemic Stroke	Parallel	Fugl-Meyer Assessment of Upper Extremity Function	NA	Chinese Clinical Trial Registry	Prospec tive registrat ion
**Jialing Wang**	2024	ChiCTR 2400081 810	China	Treatment in the experimental group: visual motor imagination combined with routine rehabilitation therapy	Stroke	Parallel	Fugl-Meyer Assessment-Upper Extremity, FMA-UE); Average value of Oxy-Hb concentration changes detected by fNIRs	NA	Chinese Clinical Trial Registry	Retrosp ective registrat ion
**Maria I. Lapid, M.D.**	2025	NCT068 97670	United States	NA	Mood Disorders and Mild Cognitive Impairme nt	Observa tional	Changes in oxyhemoglobin [Time Frame: Baseline, 60 minutes]	https://clin icaltrials.g ov/ct2/sho w/NCT06 897670	International Clinical Trials Registry Platform	Recruiti ng
**Qu Mengjian**	2025	ChiCTR 2500096 992	China	Robot-assisted training	Stroke	Parallel	Fugl-Meyer Assessment for Upper Extremity;	https://ww w.chictr.o rg.cn/sho wproj.htm l?proj=25 8324	International Clinical Trials Registry Platform	Pending
**Daria Petrova, PhD**	2025	NCT068 06397	Russia n Federa tion	Diagnostic Test: Stimulus response task	Stroke	Observa tional	Hemodynamic changes after stroke [Time Frame: 14 d]	https://clin icaltrials.g ov/ct2/sho w/NCT06 806397	International Clinical Trials Registry Platform	Recruiti ng
**Na Ye**	2025	ChiCTR 2500096 562	China	High-frequency treatment group: High-frequency	Ischemic Stroke	Parallel	FMA-UL	https://ww w.chictr.o	International Clinical Trials	Pending

				rTMS (5 Hz); low-frequency treatment group: Low-frequency rTMS (1 Hz); Sham Stimulation Group: Sham Stimulation; normal control group: none.				rg.cn/showproj.html?proj=257247	Registry Platform	
**Ling Hua**	2025	ChiCTR2400092803	China	Healthy group: verbal and visual spatial working memory tasks; Brain injury group: verbal and visual spatial working memory tasks	Stroke, traumatic brain injury	Parallel	Forward Digit Span Test; The visual patterns test;	https://www.chictr.org.cn/showproj.html?proj=244414	International Clinical Trials Registry Platform	Pending
**Hong Kong Metropolitan University**	2025	NCT06971510	Hong Kong	Device: Exoskeleton Training Other: Conventional Physical Therapy	Spinal cord injury	Allocation Randomized Interventional Model Parallel Assignment Interventional Model Description:	Duration of a turn; Number of steps to a turn [Time Frame: baseline, session 12 (6 wk), session 24 (12 wk), 1-mon follow-up]	NA	Clinical Trials	Active, not recruiting

						1) Exoskeleton training group, 2) conventional training group; 3) usual care group Masking Single (Investigator) Primary Purpose Treatment				
**Centre Hospitalier Régional d'Orléans**	2025	NCT06218563	France	Vibration-induced illusion of movement	Stroke	Interventional Model Single Group Assignment Masking :Open Label	Mean change in the concentration of oxyhemoglobin during the task;<br>Mean change in the concentration of deoxyhemoglobin during the task [Time Frame: Day 0]	NA	Clinical Trials	Recruiting
**IRCCS San Raffaele**	2025	NCT06868160	Italy	tDCS	Parkinson's disease	Allocation Randomized Interven	Total execution time of Timed up and go test with cognitive dual-task (TUG-COG) [Time Frame: Baseline, 6, 14, 20, and 28	NA	Clinical Trials	Not yet recruiting

tional Model Parallel Assignment Interventional Model Description: Every patient will undergo two rounds of training (6 weeks) with a 8-week washout period in between. Patients will be randomized in two parallel groups: 1/3 of the	wk]

patients will receive two rounds of rehabilit ation + SHAM stimulati on; 2/3 of the patients will receive two rounds of rehabilit ation + non-inv asive STIMU LATIO N.

Patients in the STIMU LATIO N group will be further randomi zed in two

groups both receiving tDCS stimulation combined with tsDCS or SHAMtsDCS in a crossover design: one group will receive a first round of rehabilitation + tDCS + tsDCS followed by a round of rehabilitation + tDCS + SHAM tsDCS; the other group will

receive the same treatment in an inverted order.

Masking Double (Participant Outcomes Assessor) Masking Description: The assessor blinded will be neurologists, neuropsychologists and physiotherapists who will perform the evaluations at each

		time point.<br><br>Patients will not know if they are receiving sham or real stimulation.<br><br>Primary Purpose Supportive Care	
